# Mutations in *LRRC50* Predispose Zebrafish and Humans to Seminomas

**DOI:** 10.1371/journal.pgen.1003384

**Published:** 2013-04-11

**Authors:** Sander G. Basten, Erica E. Davis, Ad J. M. Gillis, Ellen van Rooijen, Hans Stoop, Nikolina Babala, Ive Logister, Zachary G. Heath, Trudy N. Jonges, Nicholas Katsanis, Emile E. Voest, Freek J. van Eeden, Rene H. Medema, René F. Ketting, Stefan Schulte-Merker, Leendert H. J. Looijenga, Rachel H. Giles

**Affiliations:** 1 Department of Medical Oncology, University Medical Center Utrecht, Utrecht, The Netherlands; 2 Center for Human Disease Modeling, Department of Pediatrics, and Department of Cell Biology, Duke University Medical Center, Durham, North Carolina, United States of America; 3 Department of Pathology, Erasmus MC, University Medical Center Rotterdam, Daniel den Hoed Cancer Center, Josephine Nefkens Institute, Rotterdam, The Netherlands; 4 Hubrecht Institute, KNAW and University Medical Center Utrecht, Utrecht, The Netherlands; 5 Department of Pathology, University Medical Center Utrecht, Utrecht, The Netherlands; 6 Department of Nephrology and Hypertension, University Medical Center Utrecht, Utrecht, The Netherlands; University of Utah, United States of America

## Abstract

Seminoma is a subclass of human testicular germ cell tumors (TGCT), the most frequently observed cancer in young men with a rising incidence. Here we describe the identification of a novel gene predisposing specifically to seminoma formation in a vertebrate model organism. Zebrafish carrying a heterozygous nonsense mutation in Leucine-Rich Repeat Containing protein 50 (*lrrc50* also called *dnaaf1*), associated previously with ciliary function, are found to be highly susceptible to the formation of seminomas. Genotyping of these zebrafish tumors shows loss of heterozygosity (LOH) of the wild-type *lrrc50* allele in 44.4% of tumor samples, correlating with tumor progression. In humans we identified heterozygous germline *LRRC50* mutations in two different pedigrees with a family history of seminomas, resulting in a nonsense Arg488* change and a missense Thr590Met change, which show reduced expression of the wild-type allele in seminomas. Zebrafish *in vivo* complementation studies indicate the Thr590Met to be a loss-of-function mutation. Moreover, we show that a pathogenic Gln307Glu change is significantly enriched in individuals with seminoma tumors (13% of our cohort). Together, our study introduces an animal model for seminoma and suggests LRRC50 to be a novel tumor suppressor implicated in human seminoma pathogenesis.

## Author Summary

Testicular Germ Cell Tumors are frequently occurring tumors, affecting 1 in 500 individuals. Of this diverse group, the subtype seminoma is most prevalent and is the most common tumor type found in men aged 20–40 years of age. In contrast to other frequently occurring tumor types, there is very little information on the genetic components that form risk factors for seminoma. In this study we describe the unexpected finding that zebrafish carrying a heterozygous mutation in the *lrrc50/dnaaf1* gene have a high incidence for testicular germ cell tumor formation. Detailed analysis suggests that these tumors resemble human seminoma. We therefore analyzed this gene in a subset of human seminoma samples and recovered mutations that were subsequently demonstrated to prohibit protein function. Seminomas were also previously found in family members of these patients, suggesting that a genetic component is the underlying cause. We thus identified a novel gene that can be considered a risk factor for human seminoma, and we describe an animal model system that is valuable for further seminoma research.

## Introduction

Human testicular germ cell tumors (TGCT) [MIM 613190] affect 1 in 500 Caucasian men. Current clinical classification recognizes five main subcategories with diverse clinical manifestations, genomic constitution and pathology [1]. TGCTs have their origin in the oncogenic counterparts of cells derived from the embryonic stage of the germ lineage. So-called Type II TGCTs, which are the most predominant tumor types diagnosed in Caucasian men aged 20–40, derive from primordial germ cells (PGC)/gonocytes that have become blocked in their maturation and form *carcinoma in situ* (CIS) cells [1]. Depending on incompletely understood factors these form the uniform pathology seminoma, which is considered the default tumor type developing from CIS. Alternatively, CIS cells can also develop into non-seminoma, a more mixed tumor spectrum that includes characteristics of undifferentiated stem cells, which are expected to arise in part through epigenetic reprogramming. An overview of the development of various TGCT subtypes is provided in Figure S1 [1], [2]. The incidence for seminomas, representing the major component of TGCT type II, is rising [1]; nevertheless, there are sparse data describing genetic alterations functionally contributing to seminoma development, and previously described mammalian models did not have sufficient analogy to human seminoma [1].

In recent years, zebrafish have emerged as an established and tractable vertebrate animal model that contributes to current oncology research [3]. Many basic developmental processes are well conserved from fish to mammals, including germ line development [4] and earlier described TGCT isolated from zebrafish seem to resemble human TGCT characteristics [5]. We previously described a loss-of-function mutation in zebrafish *lrrc50^Hu255h^*, of which homozygous mutants display the ciliopathy phenotypes of primary ciliary dyskinesia (PCD) (CILD1; MIM 244400) in humans [6]. An essential function in proper cilia function has now been attributed to LRRC50 across eukaryotic taxa in organisms ranging from *Chlamydomonas* and zebrafish to humans (CILD13; MIM 613190) [6]–[9]. Here, we describe the susceptibility to tumor formation of heterozygous *lrrc50^Hu255h^* zebrafish and suggest a tumor suppressor role for *LRRC50* (alias DNAAF1; dynein assembly factor 1) in the specific development of the TGCT subtype seminoma in both zebrafish and man.

## Results

### Heterozygous zebrafish *lrrc50^Hu255h^* are predisposed to testicular tumor formation

**Figure 1:**
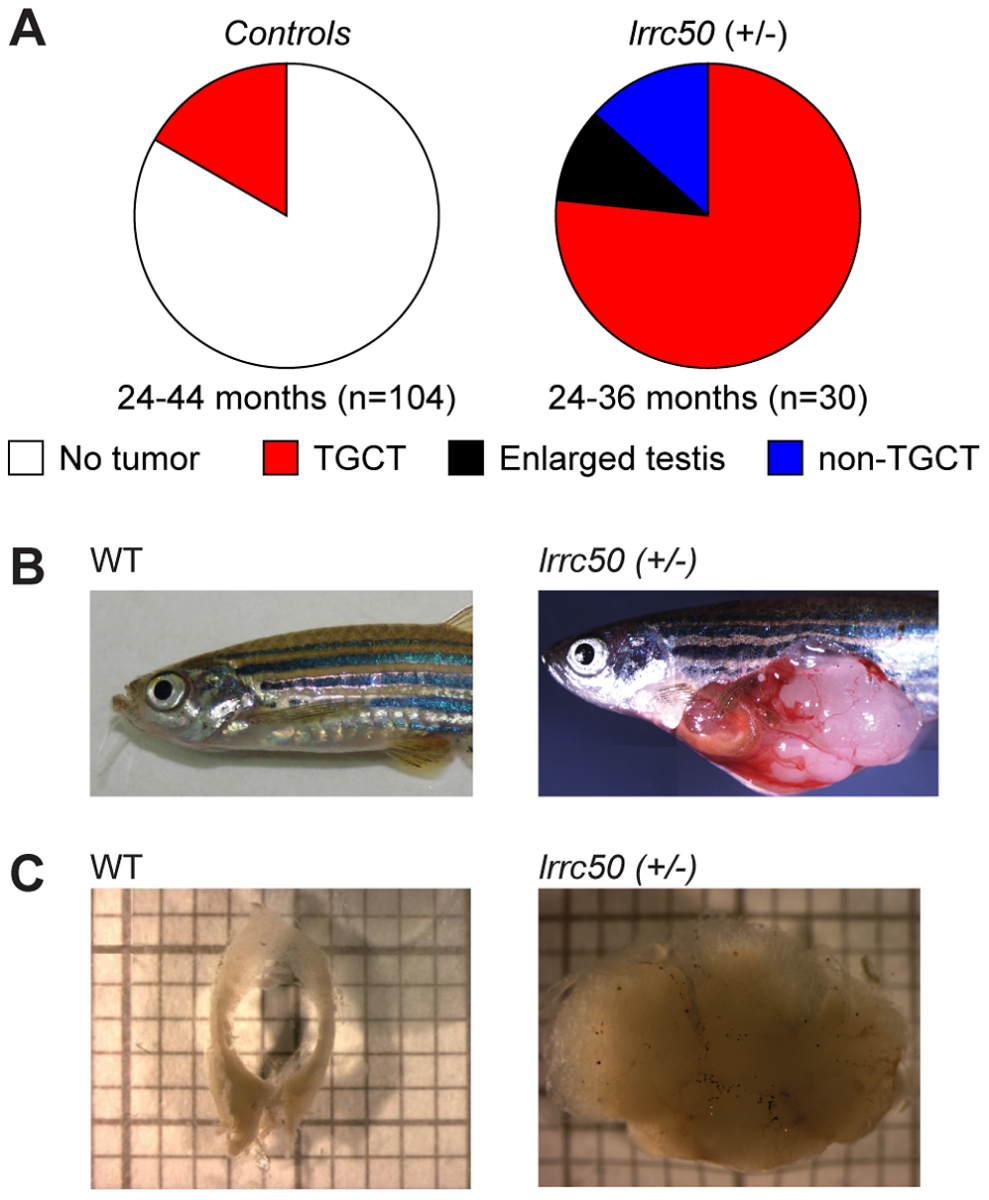
Heterozygous *lrrc50^Hu255h^* zebrafish are predisposed to testicular tumor formation. (A) Incidences of tumors extracted from randomly selected male controls derived from ENU-mutagenesis (n = 104) and heterozygote male *lrrc50^Hu255h^* (n = 30) zebrafish between 24–44 months of age are summarized in pie charts. Tumor formation presented as both TGCT and non-TGCT (sporadic tumors in somatic tissues) in the *lrrc50^Hu255h^* cohort (90%) is significantly elevated from TGCT (16.3%) formation in the controls (*P*<0.0001). No alternative tumor types were noticeable in the control group. (B) An age-matched wild-type zebrafish compared to a TGCT bearing *lrrc50+/−* zebrafish (skin around tumor removed; merge of three images). (C) Wild-type testes are composed of two tubular arms forming the paired gonad. The tissue architecture is severely disrupted in the tumor.

Whereas homozygous *lrrc50* (−/−) mutants develop lethal defects during larval development due to severe ciliopathy phenotypes [6], [10], heterozygous *lrrc50^hu255h^* (+/−) zebrafish develop into adulthood without apparent defects. Noticeably, we observed unexpectedly high tumor prevalence in the male population (n = 30) during the second and third year of life, with a penetrance exceeding 90% (Figure 1A). Testes are the predominant tissue for tumor formation (Figure 1B), although sporadically tumors were also observed in other tissues (Figure 1A, and non-TGCT examples in Figure S2A, S2B). Histological analyses (n = 11) indicate that females develop no gonadal abnormalities (Figure S2C). The recovered tumors display uniform loss of macroscopic normal testicular architecture (Figure 1C). The tumors are well encapsulated and do not appear to be metastatic; upon tumor isolation no abnormal visceral organs were observed. Analysis of 104 randomly selected age-matched male zebrafish (24–44 months old) that had similarly been generated through *N*-ethyl-*N*-nitrosourea (ENU) mutagenesis showed a common background level (16.3%) of TGCT formation (Figure 1A).

### Zebrafish *lrrc50* testicular tumors appear analogous to human seminoma

**Figure 2:**
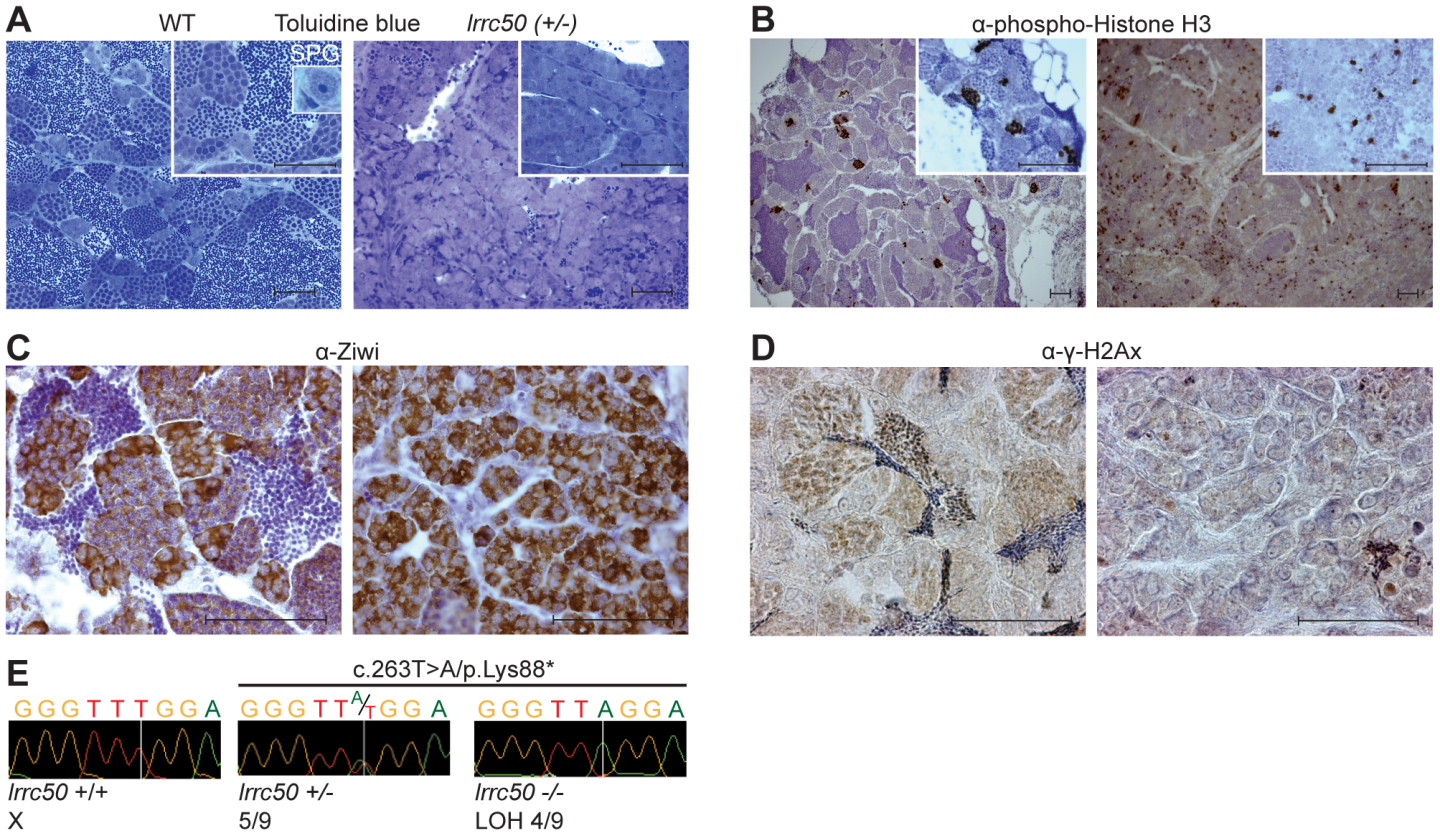
Characterization of *lrrc50^Hu255h^* zebrafish TGCT suggests analogy to human seminoma. (A–D) Histological characterization of wild-type testis (left panels) and *lrrc50^H255h^* tumors (right panels). Two magnifications shown A, B (largest in insert) all scale bars; 50 µm. The characterization indicates the presence of predominantly early germ cells and loss of differentiated germ cells in the tumors. (A) Morphological tissue analysis of toluidine blue stained sections indicates the presence of all stages of spermatogenesis in normal tissue (extensively described in Figure S3A), and shows a dramatic loss of differentiated germ cells in the tumor. (B) IHC characterization with proliferation marker α-*phospho*-HistoneH3 (pH3) shows synchronously dividing cell-clusters in normal tissue, indicative of differentiated germ cells. Early SPG is the only germ cell that can divide as a single cell, and the tumors show mostly single proliferating cells. Increased pH3 staining suggests the tumor tissue is highly proliferative (quantified in Figure S4A). (C) IHC using α-Ziwi (strong cytoplasmic expression). In normal tissue, Ziwi expression is restricted to early SPG and gradually and diffusely lost differentiated germ cells. With the exception of somatic tissue, the tumors are almost completely composed of early SPG. (D) IHC with meiosis marker α-γ-H2Ax shows normal tissue that is composed of various stages of differentiation, whereas these are predominantly absent in tumor tissue. (E) Chromatograms of WT zebrafish, heterozygote *lrrc50^Hu255h^* and *lrrc50^Hu255h^* tumors. We observe a loss of the remaining wild-type allele c.263T>A/p.Lys88* in 44.4% of the tumors (LOH).

Wild-type zebrafish adult testes are composed of few SPG/gonocytes and large numbers of differentiated germ cells or mature sperm (Figure 2A, high resolution image in Figure S3A) [11]. The recovered tumors generally present severely reduced or total absence of end-stage differentiated germ cells and an increase of cells morphologically resembling early spermatogonial cells (SPG) (Figure 2A). Three testes from fish without externally evident tumors (Figure 1A) morphologically contained increased numbers of both pre- and post-meiotic cells that most likely represents hyperplasia, but might additionally suggest that population expansion precedes tumorigenesis (Figure S3B) [12]. Differentiated spermatogonia typically remain interconnected through a stabilized intercellular bridge (forming a syncytium), licensing unbound exchange of cytoplasmic components resulting in population synchronization [13]. Immunohistochemistry (IHC) with mitotic marker *phospho*-Histone H3 (pH3) in wild-type testis occasionally stains single stem cells -the only germ cell type dividing as a single cell- and marks clutches of synchronously dividing differentiated cells, while the tumors are predominantly composed of single proliferating cells (Figure 2B). Upon quantification (Figure S4A), we observed significantly increased numbers of individual proliferating cells (*P* = 0.0025, non-parametric Mann-Whitney test), suggesting that these tumors are highly proliferative and enriched for single cells. The zebrafish tumors consist of a morphologically uniform tumor cell population, which is most analogous to human seminoma. A human seminoma-specific marker is HIWI [14], whose zebrafish ortholog Ziwi is described [15] to have similar elevated expression in early germ cells and reduced diffuse expression in differentiated germ cells (Figure 2C). Ziwi IHC on *lrrc50^Hu255h^* tumors shows strong staining in the majority of cells with the exception of somatic tissue. Staining with meiosis marker γ-H2Ax shows various stages of differentiated germ cells in wild-type, but almost complete absence in the tumors, indicating a pre-meiotic population of cells (Figure 2D, 2E) [16]. More in-depth studies would be useful to correlate the tumor characteristics of our zebrafish tumors with human seminomas, such as staining with the additional TGCT markers for early germ cells Nanog and Oct3/4, in order to affirm a seminoma analogy more accurately. Nevertheless, the zebrafish tumors have a severe early germ cell differentiation defect, and based on both the morphology and the combination of the various histological analyses of *lrrc50^Hu255h^* tumors we suggest an initial specific classification as seminoma is supported.

### Zebrafish seminomas display *lrrc50* LOH

We next interrogated the somatic loss of the wild-type *lrrc50* allele in zebrafish tumorigenesis by genotyping the Hu255h (c.263T>A/p.Lys88*) nonsense mutation and LOH was found in 44.4% of tumors (n = 4/9) (Figure 2F); direct sequencing of the coding regions of the *lrrc50* locus revealed no additional mutations. Tumors lacking evident LOH could potentially reflect the presence of wild-type tissue and/or variable tumor progression. When tumor genotypes and tumor progression are compared based on morphology and Ziwi expression, LOH strongly correlates with samples where sperm content is relatively low and there is an abundance of early germ cells (Figure S4B). Alternatively, undetected inactivating lesions (e.g. promotor/intronic sequences, large chromosomal deletions), epigenetic alterations, or unrelated background tumors could obscure genotypic analysis. Although we cannot exclude an underlying haploinsufficient mechanism, we suggest that zebrafish *lrrc50^hu255h^* seminoma progression is consistent with biallelic inactivation.

### Mutations in *LRRC50* are associated with human seminoma

**Figure 3:**
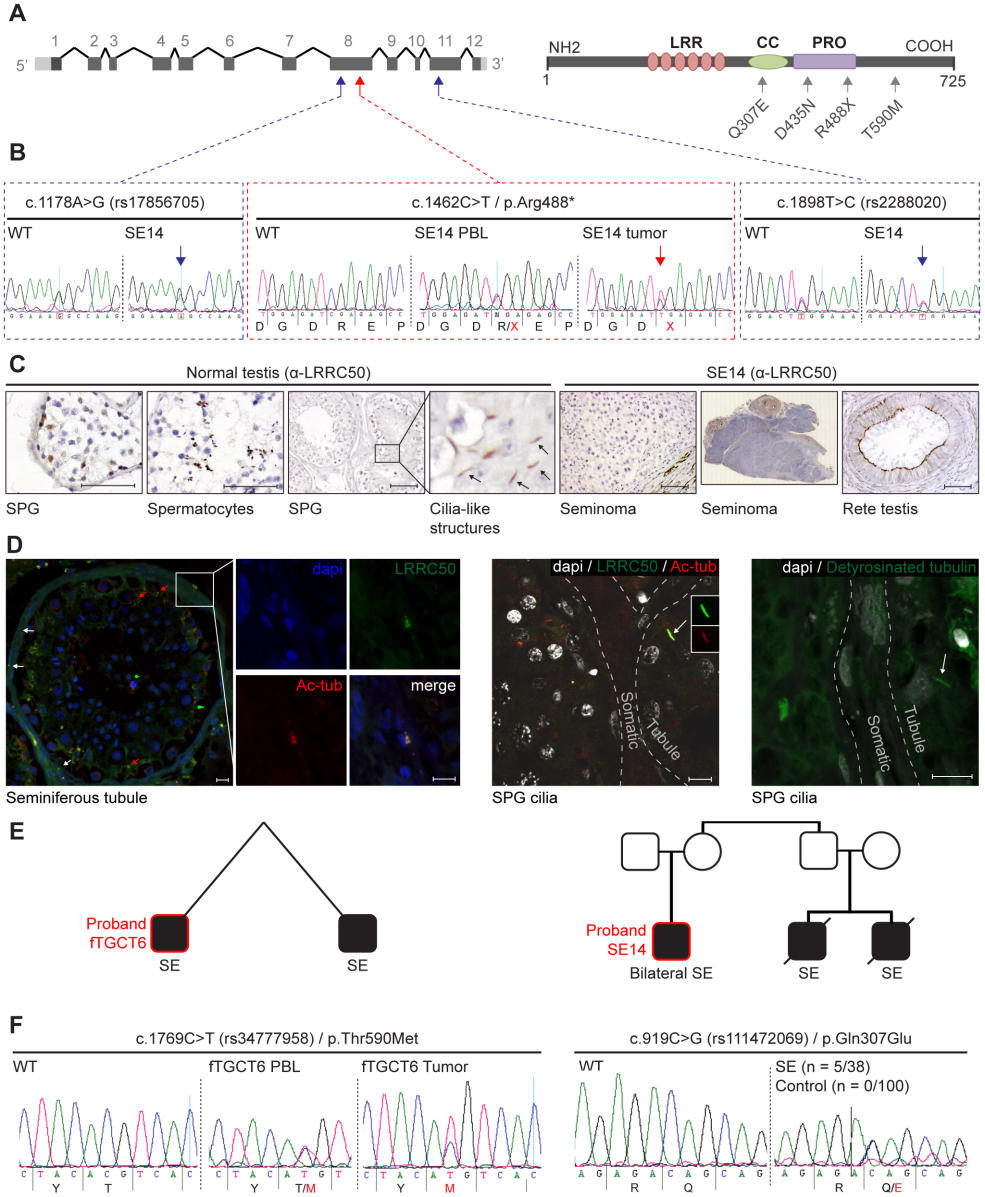
Genetic analysis of *LRRC50* in human seminoma samples. (A) *LRRC50* spans 12 exons on chromosomal arm band 16q24.1. All recovered genetic variations (see Table 2) are indicated in the LRRC50 structure. (B) Chromatogram of SE14-PBL reveals a germline heterozygous nonsense c.1462C>T/p.Arg488* (TMP_ESP_16_84203896) mutation (red arrow in A). Bilateral SE14-seminomas (one sample shown) show a constitutively stronger mutant peak. Analysis of up- and downstream SNPs in closest proximity (blue arrows in A), c.1178A>G/rs17856705 in exon 8 and c.1898T>C/rs2288020 in exon 11, render equivalent peak intensities, indicative of a regional LOH in the tumor. (C) IHC for LRRC50 stains SPG and spermatocytes in normal testis, interestingly; structures reminiscent of cilia in SPG are observed. The SE14 tumor shows complete loss of LRRC50 protein expression. Motile cilia staining of non-tumorigenic tissue (rete testis) from SE14 supports antibody specificity and proper fixation of tumor SE14 tissue. Scale bars; 50 µm. (D) IF of normal testis tissue indicates LRRC50 in cilia on germ cells lining the seminiferous tubule (red arrows) and somatic tissue of the seminiferous tubules (white arrows and insert). Early germ cells localize peripheral to the seminiferous tubule and higher resolution images (maximal projections of Z-stacks) show the presence of solitary cilia in these germ cells in more detail using cilia markers acetylated-α-tubulin (Ac-tub) and detyrosinated-tubulin, LRRC50 locates to cilia in these SPG. Scale bars; 10 µm (E) fTGCT6 and SE14 proband pedigrees. (F) Genotyping *LRRC50* in the seminoma collection (n = 38) identified one sample with a c.1769C>T/p.Thr590Met (rs34777958) allele. Additionally, heterozygous variation c.919C>G/p.Gln307Glu (rs111472069) was identified in five seminoma samples, representing a significant enrichment (*P* = 0.0013). Both variations are absent in a healthy control group (n = 100).

We next conducted *LRRC50* mutational analysis in a collection of 30 human seminomas and five spermatocytic seminomas (the latter as controls) (Table 1). We identified one individual (SE14) diagnosed with a stage-II seminoma and a contra-lateral stage I seminoma within a six-year interval; both tumors have a nonsense c.1462C>T/p.Arg488* mutation in exon 8 (Figure 3A, 3B, Table 1). Corresponding peripheral blood (PBL) revealed a heterozygous germline c.1462C>T/p.Arg488* (TMP_ESP_16_84203896) *LRRC50* mutation, which is extremely rare and identified in 0.008% (1/12,999) of chromosomes in the NHLBI Exome Variant Server (NHLBI ESP, http://evs.gs.washington.edu/EVS/). Both tumor DNA chromatograms show a consistently stronger mutant peak compared to PBL, indicating biallelic loss in at least a subset of tumor cells, or presence of non-tumorous cells (somatic tissue, lymphocytes). Analysis of SNPs in closest proximity to the mutation, rs17856705 and rs2288020, showed perfect heterozygosity in both seminomas and PBL, supportive of a localized LOH event. Accordingly, IHC staining of SE14-tumor sections with α-LRRC50 indicates no detectable protein expression, whereas IHC on rete testis (non-tumorous normal control tissue) from SE14 confirms presence of LRRC50 in ciliated somatic tissue and antibody specificity (Figure 3C). In normal testis LRRC50 is expressed in SPG and spermatocytic cells and notably appears to localize to structures resembling cilia (Figure 3C). Cilia have not previously been demonstrated in early germ cells. To investigate the cilia-like structures in more detail, immunofluorescent staining and confocal microscopy was performed on 6 normal testes obtained from autopsies of men ranging from 33–43 years of age. Not only did we observe cilia on spermatogonia upon staining for specific ciliary marker detyrosinated tubulin, but co-staining α-LRRC50 with another established cilia marker, acetylated-α-tubulin, confirms LCCR50 localization to the axoneme of the cilium. Furthermore, ciliary LRRC50 was observed in both somatic tissue cells of the seminiferous tubule (red arrows and insert) and germ cells lining the tubular epithelium (white arrows and higher resolution images) (Figure 3D).

**Table 1:** Human TGCT samples.

Sample	Primary tumor	Family tumor	Allele
SE1-SE6	SE		Gln307Glu [2×]
SE7	Bilateral SE		
SE8-SE13	SE		Asp435Asn
SE14	Bilateral SE	2 Cousins with SE	Arg488*
SE15-30	SE		Asp435Asn [2×] Gln307Glu [2×]
SS1-5	SS		Asp435Asn
fTGCT1	Bilateral SE	Family members with SE identified	
fTGCT2	Non-SE: EC, Te, YS	Father with SE	
fTGCT3	Bilateral SE	Brother with TGCT	
fTGCT4	SE	Brother with SE (fTGCT5)	
fTGCT5	SE	Brother with SE (fTGCT4)	
fTGCT6	SE	Monozygous twin with SE	Thr590Met
fTGCT7	Non-SE: EC	Brother with SE	
fTGCT8	Bilateral SE	Cousin with TGCT	Gln307Glu
fTGCT9	Non-SE: EC, Te, YS	Monozygous twin with TGCT	
fTGCT10	Non-SE: EC, imTe, YS, Ch	Monozygous twin with TGCT	
fTGCT11	Bilateral SE	Brother with SE	
fTGCT12	Bilateral SE	2 Brothers with TGCT	
fTGCT13	SE+non-SE: EC, Te, YS	Father with non-SE	
fTGCT14	SE+non-SE: EC	Father with non-SE	
fTGCT15	Bilateral SE+non-SE: Te, EC, YS	Father and 2 cousins with TGCT	

Genotypic analysis was performed on human TGCT samples (n = 51); a total of 30 seminomas (SE) and five spermatocytic seminomas (SS) were initially analyzed. These samples were expanded with 15 fTGCT samples, including eight seminomas, which were isolated from patients known to have a familial background of seminomas. Non-SE = non-seminoma, EC = embryonic carcinoma, (im)Te = (immature) teratoma, YS = yolk sac tumor, Ch = choriocarcinoma. Patients carrying alleles identified in this study are indicated.

Pedigree analysis of the proband SE14 (Figure 3E) revealed two first cousins who died of seminoma as teenagers (samples unavailable), suggestive of an underlying genetic predisposition. The presence of familial TGCTs and previous diagnosis with seminoma are risk factors for the formation of contra-lateral TGCT [17]. Therefore, we screened *LRRC50* sequences in 15 TGCTs with a known familial incidence (Table 1). We identified one sample (fTGCT6) harboring a heterozygous c.1769C>T/p.Thr590Met missense mutation (Figure 3A, 3F, Table 1), predicted to be damaging by PolyPhen-2 [18]. This is a rare SNP (rs34777958) not detected in an ethnically matched male control group (n = 100), and is present in 1.15% (150/12,850) of chromosomes in the NHLBI ESP. Additionally, genotype data in dbSNP137 showed that Thr590Met is not present in homozygosity in the NHLBI ESP cohort suggesting that it is a deleterious change likely under purifying selection. Patient fTGCT6 and his monozygotic twin brother both developed seminoma. Again, the mutant chromatogram is stronger in the tumor than in PBL, suggesting LOH. In the total seminoma population (n = 38) we identified a significantly enriched, heterozygous, conserved mutation, c.919C>G/p.Gln307Glu (n = 5, 5/76 alleles, *P* = 0.0013, Fisher's exact test), potentially associated with seminoma as it is absent in a healthy male control group (n = 100, 0/200 alleles) (Table 1, Table 2 and Figure 3A, 3F). We sequenced the entire coding sequences of the *LRRC50* gene (primer sequences provided in Table S2) in these samples but observed no additional exonic mutations, apart from frequently occurring SNPs without predicted pathogenicity, excluding compound heterozygosity. The Gln307Glu allele is present 2.9% (375/12625) in the ESP cohort, however we cannot exclude the possibility that some ESP males may have been affected with seminomas.

**Table 2:** Genetic variation of *LRRC50* in human seminomas.

Variation	Protein	Control group (n = 100)	Seminoma (n = 38)	dbSNP
c.919C>G	p.Gln307Glu	0	5	rs111472069
c.1303G>A	p.Asp435Asn	4	4	rs149158199
c.1462C>T	p.Arg488*	0	1	TMP_ESP_16_ 84203896
c.1769C>T	p.Thr590Met	0	1	rs34777958

Genotyping of seminoma (n = 38) samples and a control group (n = 100) revealed several variations. c.919C>G/p.Gln307Glu (rs111472069) is a heterozygous mutation significantly more frequently observed (*P* = 0.0013) in the seminoma group (5/38 samples) and absent in the control group (0/100 samples). We identified a heterozygous mutation c.1303G>A/p.Asp435Asn (rs149158199), however this mutation is, seemingly less predominant, also identified in the control group and was also identified in one spermatocytic seminoma patient. The known variation c.1769C>T/p.Thr590Met (rs34777958) was identified in one seminoma patient with a monozygotic twin brother that had also developed a seminoma. Both Gln307Glu and the Thr590Met variants are shown to be functional nulls in this study. The positions of the variations are indicated in the protein structure in Figure 3A.

### Missense LRRC50 alleles identified in seminomas are functional nulls

**Figure 4:**
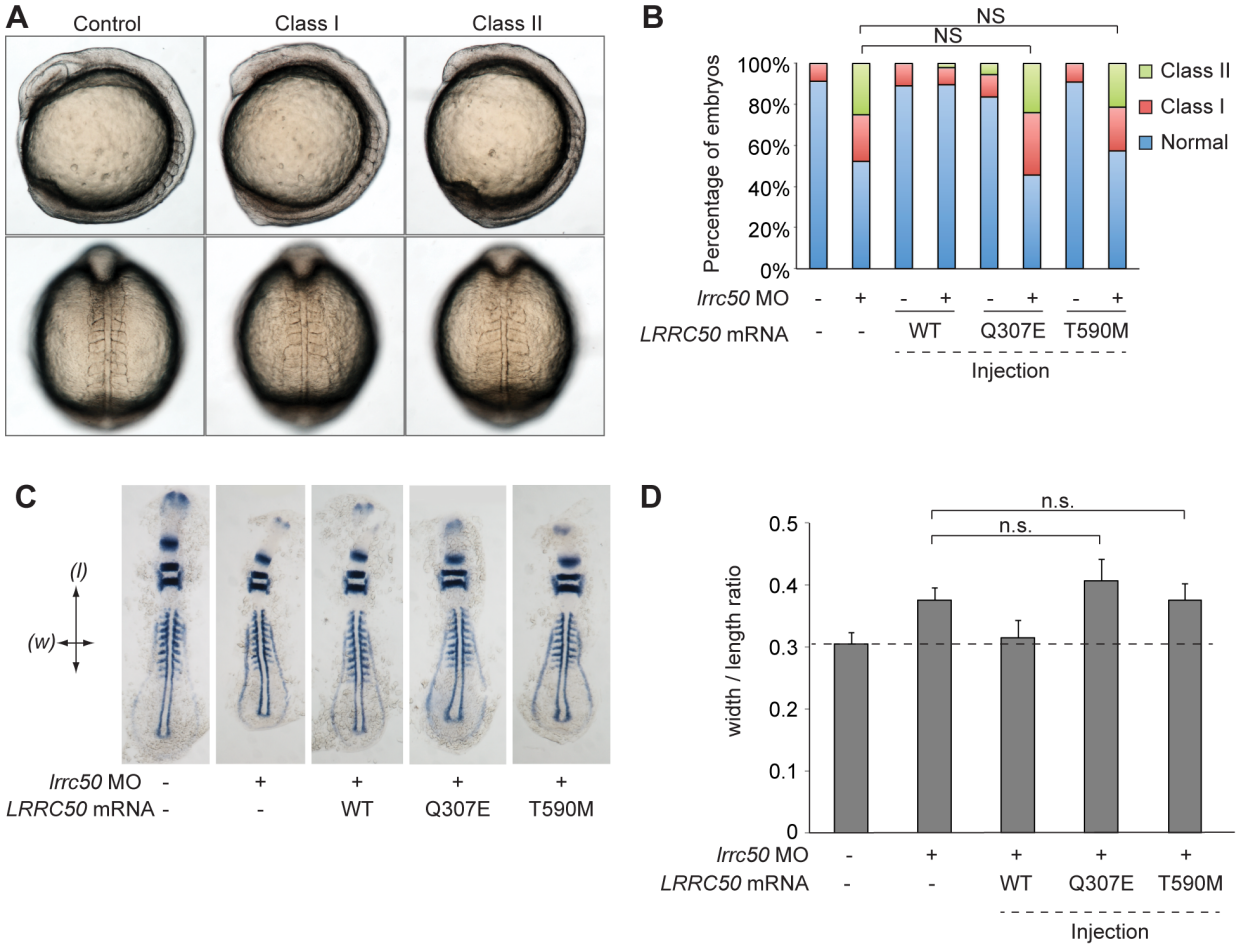
LRRC50 missense changes are functional nulls in a zebrafish model of development. (A) Live embryo images of *lrrc50* morphants. Morpholino (MO) mediated suppression of *lrrc50* gives rise to gastrulation defects in mid-somitic zebrafish embryos that can be categorized according to previously established objective scoring criteria [19], [57]. Representative lateral and dorsal views are shown (top and bottom panels respectively). (B) Scoring of *in vivo* complementation; WT embryos were injected with MO and/or human mRNA and scored at the 8–9 somite stage according to phenotypes shown in panel A. Gln307Glu and Thr590Met are not significantly different (NS; c^2^) from MO alone suggesting that both are functional nulls, n = 44–79 embryos/injection repeated twice with masked scoring. (C) Representative images of flat mounted *in situ* hybridized (ISH) zebrafish embryos labeled with a cocktail of *krox20*, *myoD*, and *pax2a* riboprobes. Arrows indicate measurement parameters for morphometric analyses shown in panel D; length (*l*) was measured as the anterior-posterior distance from the first to the last appreciable somite; width (*w*) was measured as the distance spanning the lateral tips of the fifth somites counted from anterior to posterior. (D) Morphometric quantification of Gln307Glu and Thr590Met gastrulation defects. Images of flat-mounted embryos age-matched at 9 somites were measured in two dimensions (as shown in panel C); the ratios of medial-lateral (width; *w*) versus anterior-posterior (length; *l*) measurements are shown for randomly chosen embryos from live scoring experiments (panels A and B) for ISH. *LRRC50* Gln307Glu and Thr590Met do not produce a significant rescue in comparison to MO alone (NS; t-test), corroborating the *in vivo* scoring results in panel B (n = 9–13 embryos/injection). Error bars indicate standard deviation of the mean.

To test the functional consequences of Thr590Met and Gln307Glu on protein function, an *in vivo* complementation approach was employed in zebrafish. Since maternally-derived WT *lrrc50* mRNA can still be detected in *lrrc50^hu255h^* mutants early in development [6], we opted to use transient morpholino (MO)-induced suppression designed to block maternal and embryo-derived *lrrc50* translation. We have previously shown that in addition to the PCD and renal cystic phenotypes of *lrrc50* mutants, transient MO-induced suppression of *lrrc50* gives rise to gastrulation phenotypes in mid-somitic embryos, which can be rescued by wild-type (WT) capped human *LRRC50* mRNA (Figure 4A, 4B, scoring shown in Table S1) [19]. Here, we test *LRRC50* mRNA harboring either Thr590Met or Gln307Glu missense mutations to rescue MO-induced gastrulation defects. Whereas co-injection of WT message with MO results in a significant rescue in comparison to MO alone (*P*<0.0001; c^2^), embryo batches injected with either missense change resulted in no significant rescue suggesting that both variants are functional nulls in this assay (Figure 4B, scoring shown in Table S1). Importantly, mutant *LRRC50* message injected alone did not produce a significantly different phenotype from that of WT mRNA. To corroborate these findings, two dimensional morphometric analysis of the gastrulation defects are conducted. We labeled anatomical landmarks of 9-somite stage embryos with a cocktail of *krox20*, *pax2*, and *myoD* riboprobes, and measured the ratio of the width spanning the fifth somite counted from the anterior end of the embryo versus the length from the first to the last appreciable somite (Figure 4C, 4D, scoring shown in Table S1). Consistent with the *in vivo* scoring data, the measurements capturing the gastrulation defects of *lrrc50* morphants were statistically indistinguishable from those of either of the two mutant rescue batches (n = 9–13 embryos/batch), substantiating further the notion that both changes are detrimental to protein function.

### LRRC50 is dynamically localized and expressed in a cell-cycle-dependent fashion

**Figure 5:**
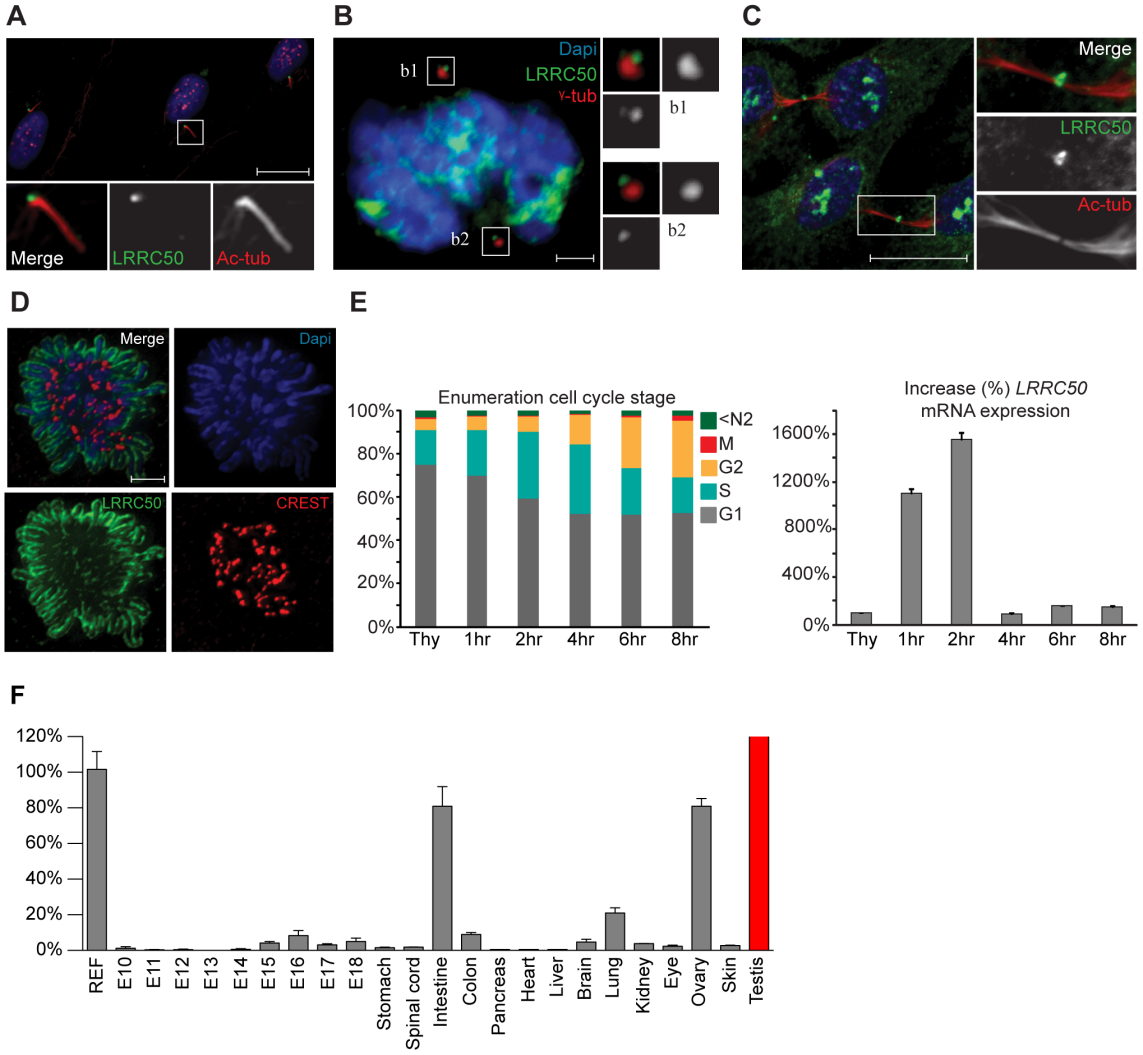
Localization and expression of LRRC50. LRRC50 antibody ab75163 (reproducible with alternative LRRC50 antibodies) shows a dynamic, cell cycle dependent, distribution in RPE-hTERT cells (representative for other tested mammalian cell lines). Panels represent LRRC50 (green) at various stages, counterstained with DAPI (blue) and acetylated-α-tubulin (Ac-tub, red). Optical sections in A–C are 3 µm. (A) In ciliated serum-starved cells, LRRC50 localizes to the peripheral centrosome/basal body region dorsal of the axoneme (red). Scale bar 10 µm. (B) In mitotic cells, LRRC50 remains associated with the duplicated centrosomes, as indicated with γ-tubulin (red). Inserts b1,2 demonstrate a peri-centrosomal localisation. Scale bar 2 µm. (C) Temporal localization to the midbody in cytokinesis. Scale bar 10 µm. (D) During mitosis LRRC50 dynamically associates with condensed chromosomes (extensively described in Figure S5B). Counterstain CREST (red) marks kinetochores. Image is a maximum intensity projection of deconvoluted stacks. Scale bar 2 µm. (E) Dynamic *LRRC50* mRNA expression (correlating with dynamic localization) with error bars as standard deviation. T47D cells synchronized at the G1/S transition with thymidine show a strong *LRRC50* transcript up-regulation upon release, specifically in the cell population entering early S-phase (see also Figure S6A). Intriguingly; although the protein remains stable during mitosis (shown in D) the transcript is rapidly down-regulated, and restored to basal levels. (F) Expression profiling of *LRRC50* mRNA expression with error bars of standard deviation in a library of mouse cDNA tissues normalized to full mouse reference pool. Testis expression shown in red as the expression level (>26.000%) strongly exceeds the normalized value.

Current knowledge of LRRC50 function is limited to ciliary motility and ciliogenesis [6]–[10], [ 20], but lacks molecular detail. We therefore used immunofluorescence (IF) and four polyclonal LRRC50 antibodies from different companies to characterize the protein. In serum-starved ciliated RPE-hTERT cells, we confirmed endogenous localization to the basal body (Figure 5A) [6], [7], [20], [21]. Moreover, LRRC50 maintains centrosomal association throughout the cell cycle and temporarily localizes to the midbody (Figure 5B, 5C). Midbody localization is manifested by multiple centrosome/basal body-related proteins [22]–[25], and LRRC50, structurally and dynamically, closely resembles a specific subset of LRR-proteins sharing multiple characteristics and cellular localization patterns (Figure S5A) [26]. Intriguingly, we observed that LRRC50 also associates with condensed chromosomes (Figure 5D), reminiscent of the dynamic localization exhibited by perichromosomal sheath proteins (Figure S5B) [27]. Similarly, *LRRC50* mRNA expression is subjected to a stringent cell cycle regulation (Figure 5E, FACS profiles presented in S6A). Analysis of *Lrrc50* mRNA expression in a mouse cDNA library of developing embryonic stages and adult tissues shows high expression levels in the ciliated tissues testis, lung and ovary, but also in highly proliferating intestinal tissue (Figure 5F). In RPE-hTERT cells we observed increased *LRRC50* expression upon serum starvation, which promotes cell cycle exit and initiation of ciliation [28] (Figure S6B). Collectively, the expression and localization data confirms the ciliary role of LRRC50, but suggests there may be additional functions other than cilia regulation (Figure S6C, summarizing model in Figure S7).

## Discussion

In this manuscript we characterize a novel vertebrate model for human seminoma associated with biallelic inactivation of *lrrc50* in at least 44.4% of tumors tested. We translated this finding to humans and identified pathogenic germline *LRRC50* mutations in two human seminoma pedigrees that had at least partially lost expression of the wild-type allele in their tumors. In addition, a significant enrichment of a pathogenic Gln307Glu change in sporadic seminomas (13% of cases) was identified, which is absent from a healthy control population, and low in the general population. Although functional evidence indicates that Gln307Glu is detrimental to protein function, population frequency data suggests that in homozygosity, it is likely not sufficient to cause PCD when inherited in the germline (0.001% of the ESP cohort is homozygous for this change). However, in the context of seminoma, this change potentially represents a significant genetic risk factor.

Wild-type zebrafish have been previously described to be susceptible to the formation of TGCTs upon advanced age [29], [30], however the etiology for this is undetermined. Similarly, zebrafish derived from genetic screens typically show seminoma development upon advanced age; one large-scale study including 10,000 zebrafish determined a background percentage of 5% in two year old zebrafish, of which nearly 50% were diagnosed as seminomas [31]. One other study of genetic instability (*gin*) zebrafish mutants between 30–34 months old identified a 28% tumor incidence compared to 5% in wild-type animals, and seminomas are observed in ∼20% of the *gin* mutants [12]. In line with these results, we identified a 16.3% background incidence of seminoma formation in 104 male zebrafish derived from *N*-ethyl-*N*-nitrosourea (ENU)-based mutagenesis screens (Figure 1A). Of interest, genetic mutant zebrafish lines are typically more susceptible to develop a different tumor spectrum, most notable malignant peripheral nerve sheath tumors (zMPNST) (Figure S2A), as has been described for *p53*, ribosomal protein mutants and genomic instability mutants [32], [33]. The recently described mutant *Alk6b* zebrafish are similarly predisposed to GCT formation; of interest, tumor formation occurs earlier in life in this genetic model. The introduced loss-of-function *Alk6b* mutation fails to activate BMP target genes through downstream nuclear p-SMAD1/5/8. The authors suggest that haploinsufficiency is a likely mechanism for the observed tumor phenotype, which is consistent with the observed latency of heterozygous compared to homozygous mutants, but possible LOH events cannot be excluded [34]. Here, we observed a tumor penetrance exceeding 90%, which is considerably elevated from the control zebrafish assessed here, as well as in previously described studies [12], [31]. Importantly, sequencing of zebrafish seminomas identified a subset of tumors showing LOH, which is likely correlated with advanced tumor progression in these samples (Figure S4B). Both zebrafish tumors and human seminomas show biallelic loss in a subset of samples, but LOH is not evident in all samples. Despite the presence of wild type tissue, which may obscure our genetic LOH analysis in the remaining samples, we cannot rule out a potential haploinsufficient mechanism. All identified alleles we tested are loss-of-function mutations as determined by *in vivo* complementation studies and IHC, and do not show dominant negative phenotypes.

The incidence for human seminoma is rising, however currently established risk factors remain poorly described and are limited to urological and testicular developmental abnormalities such as cryptorchidism and testicular atrophy and undetermined environmental factors [35]. Since *LRRC50* mutations have been reported in PCD [8], [9], a multifaceted disease that includes infertility, we cannot exclude a correlation with impaired sperm motility, however, thus far no systematic association between PCD and seminoma has been described [36]. Of interest though, motile cilia protein *DNAH9* (MIM 603330) is frequently mutated in breast cancer (MIM 114480) [37], [38] and in line with this notion, ciliary frequencies are reduced on cells derived from breast tumors [39]. We hypothesize that the ciliary localization of LRRC50 in early germ cells (Figure 3D) and subsequent loss in tumor sections might suggest a role for this organelle in normal germ cell regulation. Loss of cilia potentially deregulates specific receptors essential for proper germ cell responses. Indeed, there is some circumferential support for this hypothesis. Somatostatin receptor 3 (Sstr3) is an established ciliary localized receptor [40] that is known to be lost in seminomas [41]. Loss of Fgf8 and Fgfr1 expression in *Xenopus* reduces cilia length [42] and expression of the human orthologs is reduced in seminoma specifically when compared to other GCTs [43]. Another interesting correlation is the aberrant expression of a *PDGFRα* transcript in CIS cells [44], and normal PDGF-AA signaling can signal through cilia, at least in fibroblasts [45]. The data presented in this manuscript would argue for an additional unique role of LRRC50 in primary cilia that is not related to its function in motile cilia: the gastrulation phenotypes described in MO-treated zebrafish are associated with primary cilia function and we have never observed these defects with PCD-associated genes previously tested. Furthermore, we have described that primary cilia formation is inhibited upon shRNA-mediated knockdown in mammalian cells [6], and show increased *LRRC50* mRNA expression upon primary cilia formation (Figure S6B). It is under debate whether cilia could have a direct contribution to tumorigenesis, but it has rather been demonstrated that well-described tumor suppressors like *VHL*, *APC*, and members of the Shh and Wnt pathway connect to cilia function [46], [47], indicating that the cilium could be implicated in tumor development. Alternatively and moreover, the intracellular localizations observed for LRRC50 and cell-cycle dependent regulation could equally well reflect a cilia-independent putative tumor suppressor function. Further studies are required to unravel the apparent diverse molecular functions of LRRC50. In what way the dysfunction or loss of *LRRC50* affects autonomous early germ cell development, and whether it induces a block in maturation, deregulates differentiation or proliferation and systematically leads to seminoma development, remains elusive.

The fundamental mechanisms underlying seminoma formation are incompletely understood, but in humans is known to involve erasure of genetic imprinting of PGC/gonocyte progenitor cells [48]. Although common characteristics have been extensively described to include aneuploidy and non-random gain and loss of chromosomes, of which gain of 12p appears important in metastatic tumors [48], little information on early initiating events is available. Causal genetic factors to seminoma development are also limited as only 1.4% of TGCT are familial cancer syndromes. Nevertheless, the familial risk factor for inherited TGCT is estimated as more than double of other familial cancer syndromes [17], appealing to the need for more genetic studies. Recent advances have implicated the KITLG/SPRY4/BAK1 and TGFβ/BMP-signaling (BMPR1B) pathways in germ cell tumor development, which include the control of differentiation, cell proliferation and apoptosis [34], [49]–[51]. Mutations in both pathway components are identified in seminomas and non-seminomas, hence differentiating factors amongst these tumors remain unknown. Furthermore, N- and KRAS activation mutations and LOH of well-accepted tumor suppressors *APC*, *p53* and *CDH-1* have been identified in both seminomas and non-seminomas [52], [53]. It is expected however, that the default pathway for CIS cell development is seminoma and that an additional event is required to induce a switch in pluripotency, leading to non-seminoma [48]. Our data identifies LRRC50 as a novel candidate and we suggest that a currently unknown tumor suppression mechanism (Figure S7) specifically predisposes to zebrafish and human seminoma development.

## Materials and Methods

### Ethical approval

All animal experiments were approved by the Animal Care Committee of the Royal Dutch Academy of Science according to the Dutch legal ethical guidelines or the Duke University Institutional Care and Use Committee. The human tumor samples used for this study were approved by an institutional review board (MEC 02.981). Samples were used according to the “Code for Proper Secondary Use of Human Tissue in the Netherlands,” developed by the Dutch Federation of Medical Scientific Societies [54].

### Zebrafish lines, tissue isolation, and histology

Heterozygote lrrc50*^Hu255h^*, vhl and randomly selected control zebrafish were isolated from a forward genetic *N*-ethyl-*N*-nitrosourea (ENU)-based mutagenesis screen as previously described and maintained according to standard protocols [6], [55]. Founder *lrrc50^+/−^* fish were outcrossed three consecutives times to wild-type lines and incrossed once to maintain the line. Prior to tissue isolation, zebrafish were euthanized by overdose of MS222. Fragments for immunohistochemistry were fixed overnight using a 4% paraformaldehyde solution containing 2% acetic acid, embedded in paraffin and sectioned at 6 µm. Fragments used for morphological analysis were fixed using 4% glutaraldehyde and embedded in glycol methacrylate (Technovit 7100, Hereaus Kulzer), sectioned at 4 µm and stained with toluidine blue. Images were captured using a Nikon Eclipse E800 equipped with a Nikon DXM1200 digital camera and Plan Apo 2×/0.1, 10×/0.45, 20×/0.75 and 40×/0.95 NA objectives.

### Tissue culture, transfections, and cell synchronization

Human RPE-hTERT and T47D cells were cultured in DMEM/F12 supplemented with 10% fetal bovine serum (Lonza), penicillin/streptomycin and ultra-glutamine (2 mM). T47D cells were subjected to a double thymidine block (10 mM, Sigma) and released for indicated times. RPE-hTERT cells were ciliated by 48 hours of serum withdrawal. Mitotic RPE-hTERT cells were trapped in nocodazole (Sigma): 1 µg/ml, collected using mitotic shake-off, swollen for 15 minutes in hypotonic (75 mM) KCl solution at 37°C and prepared for imaging using cytospin. RPE-hTERT cells were transfected with Mybbp1a-RFP using Fugene6 (Roche) according to manufacturer recommendations.

### RNA isolation, real-time PCR, and cell cycle analysis

RNA isolation was performed according to manufacturers recommendations (Qiagen, RNA isolation kit). mRNA probes; LRRC50 Hs00698399_m1 and RPL19 Hs01577060_gH, were purchased from Applied Biosystems. Real time PCR was performed in triplicate using the one-step RT-PCR kit and the 7500 system from Applied Biosystems. For mouse tissue *LRRC50* expression profiling, Clontech RNA libraries of developing embryo's and adult tissues were used. Cells for FACS analysis were fixed in ice-cold 70% ethanol, DNA content and mitotic index determined using propidium iodide and phospho-HistoneH3 according to standard conditions.

### Immunofluorescence and immunohistochemistry

Cells are fixed in either ice-cold methanol or 4% PFA with 0.1% Triton-X-100. All immuno-fluorescence stainings were performed in PBS containing 3% BSA and 5% goat serum and washed in 3% BSA in PBS. Coverslips were mounted using ProLong antifade (Molecular Probes). Primary antibodies used are α-LRRC50 (1∶100, Abcam; ab75163, 1∶100 Aviva Systems Biology; ARP53359_P050, 1∶100 Santa Cruz Biotechnology; sc-133762, 1∶100 Sigma; SAB2101390), α-acetylated α-tubulin (1∶10.000, Sigma-Aldrich T6793; clone 6-11B-1), α-CREST/ACA anti-sera (1∶10.000, Fitzgerald Industry Int.), α-γ-tubulin (1∶500, Sigma; clone GTU-88, T6557). Secondary antibodies (alexa-488, -568 and -647, various species) were obtained from Molecular Probes. Images were acquired using a Zeiss 510 Meta confocal microscope with a 63×1.3 N.A. objective and analyzed with the Zeiss LSM Meta 510 software and a Deltavision RT imaging system (Applied Precision) using 100× NA 1.4 UPlanSApo objective (Olympus) using SoftWorx software. For zebrafish immunohistochemistry, stainings were performed as described [15], [34]. Primary antibodies used were α-phosphorylated-histone H3 (1∶1000, Upstate; 06-570), α-Ziwi (1∶100, [15]) and α-γ-H2Ax (1∶200, Cell Signaling). Human testicular tissues isolated from autopsies performed at the University Medical Center Utrecht of 6 men (33–43 years of age) were used. After deparaffination and rehydration, 4 µm sections were digested in *protease XXIV* (Sigma, 0.02 mg/ml in PBS, pH 7.3, 60 minutes at room temperature) [56]. After washing and blocking in 1% BSA in PBS, primary antibodies (mouse monoclonal acetylated-α-tubulin, clone 6-11B-1 Sigma 1∶12000, rabbit polyclonal α-LRRC50, ARP53359_P050 Aviva Systems Biology, 1∶50, rabbit detyrosinated-tubulin, Millipore AB3201 1∶250) were incubated for 60 minutes at room temperature. After repeated washing in PBS, secondary antibodies are incubated for an additional 60 minutes at room temperature: goat-anti-mouse conjugated to Cy5 (Millipore, 1∶100) and goat-anti-rabbit-Cy3 (Life Technologies, 1∶100). Sections were washed again repeatedly, incubated in DAPI (diluted 1∶5000 in PBS) for 15 minutes and after a final round of washes in PBS, mounted with Fluoromount G. Stained sections are stored in the dark at 4°C until confocal imaging with a Zeiss LSM700 63× objective. IHC section (3 µm) are stained with α-LRRC50 (1∶200, Abcam; ab75163), secondary antibody used is powervision-HRP IgG (1∶200, Immunologic) and sections were counterstained with hematoxylin according to standard protocols.

### Zebrafish *in vivo* complementation assays

MO targeting lrrc50 was injected into wild-type embryos at the one to four cell stage, and rescued with capped human *LRRC50* mRNA as described [19]. We used site-directed mutagenesis to introduce missense changes Thr590Met and Gln307Glu into the WT pCS2+ *LRRC50* construct (Quick-Change Site Directed Mutagenesis kit, Agilent) and used linearized plasmid to transcribe mRNA (SP6 mMessage mMachine kit, Ambion). Live embryo scoring, RNA ISH, morphometric analyses and statistics were conducted as described [57]. Analysis is detailed in Table S1.

### Quantification of spermatogonial proliferation

To assess and compare the amount of proliferating spermatogonial stem cells between wild-type and tumor tissue, sections pH3-stainings were quantified. For analysis of the tumor samples, three photographs (20× magnification) from three different sections are used (16–20 µm distance between sections) to obtain a valid representation of various regions of the tumor. For the control samples, two sections were used given the smaller size of testis fragments. The protocol for quantification was adapted from ImageJ (http://rsbweb.nih.gov/ij/). Briefly, the difference in intensity between pH3-positive and pH3-negative background tissue was substantial enough to set the threshold by manually adjusting the background levels. Next, a binary image was created to calculate the percentage of total surface area covered with positive cells. Large clusters (>3–4 cells) of proliferating cells are removed manually to enrich for individual positive cells. To correlate the amount of proliferative spermatogonial stem cells to the total amount of tissue on a given image, the calculated surface area was set to 100% and the proliferation percentage was extrapolated. The percentage of proliferation per picture was averaged for each tissue fragment. Averages were calculated and results are represented in a box-plot. More details on the calculation are found in the statistical analysis section.

### Genotypic analysis zebrafish lrrc50^+/−^ tumors and human TGCT

DNA isolation, PCR and sequencing of tumor fragments and human samples were performed according to standard procedures. Human control samples were obtained from healthy blood donors that submitted material to the UMC Utrecht department of Medical Genetics. Primer sequences can be found in Table S2. Sequence analysis was performed using polyphred software [6] and DNASTAR Lasergene Software (http://www.dnastar.com/default.aspx).

### Statistical analysis

The number of tumors identified in the different groups (Figure 1A) were compared using a two-tailed Fisher's exact test in a 2×2 contingency table. Between 24 months and 44 months of age, male *lrrc50^Hu255h^* zebrafish (TGCT tumors n = 24, normal n = 0) compared controls (TGCT tumors n = 17, normal n = 87). The quantifications of spermatogonial stem cell proliferation (Figure S4A) were subjected to statistical analysis. As input the following data was used; the tumor averages three quantified sections per sample; whereas *vhl* (+/−) and wild type control groups averaged two sections. A non parametric Mann-Whitney test at P<0.05 was used to compare whether the averages of two groups are different, the p-value between the tumor group (n = 7) and normal wild type (n = 5) or *vhl* (+/−) (n = 4) was calculated. Wild type and the *vhl* (+/−) group are not significantly different (*P* = 0.3095, U = 7.000), both are significantly (*P* = 0.0025, U = 0.0000) different from the tumor group. Results are represented in a box-plot. To calculate the putative overrepresentation of c.919C>G/p.Gln307Glu in the seminoma cohort (Figure 3F), a two-tailed Fisher's exact test in a 2×2 contingency table was used as the data is categorical and sample sizes are large. We used n = 100 and n = 0 for the control group and n = 33 and n = 5 for the seminoma group, and determined *P* = 0.0013 confirming a significant enrichment. Expression of mRNA as described in Figure 5E; Ct values obtained were normalized and used to calculate relative expression levels in n = 4 per condition. The standard deviation (*P* = 0.05) was calculated. This was performed similarly for Figure 5F using n = 3 per condition.

## Supporting Information

Figure S1 Schematic overview of human TGCT development. Schematic representation of human testicular germ cell development (black lines) giving rise to the known tumor (orange lines) pathologies [1]. Type I GCT are not indicated, but are derived from early stages of embryonic stem cells with different characteristics. Type II GCT commonly arise from transformed, intrinsically pluripotent PGC/gonocytes forming an oncogenic counterpart known as a *carcinoma in situ* (CIS) cell. The Type II tumors can be seminomas or non-seminomas, the latter forming a more complex pathology consisting of multiple tumorigenic cell types that display characteristics of undifferentiated stem cells, as well as differentiated derivatives (the latter similarly observed in type I GCTs). In normal development, gonocytes establish the germ cell lineage in the seminiferous tubules and differentiate to spermatogonial stem cells that at some point become committed to spermatogenesis. In the seminiferous tubules, the Sertoli cell supports and co-regulates germ cell differentiation and development. Type III GCT are usually recovered from old-aged men and are thought to arise from spermatogonia, i.e., germ cell that lost their embryonic characteristics, committed to spermatogenesis, which is a more differentiated stage of spermatogenesis.
(TIF)

Figure S2 Non-TGCT zebrafish *lrrc50^Hu255h^* tumors and female gonad. (A) One fish was identified bearing a large tumor (merge of two images) located proximal to the brain, histologically resembling zebrafish malignant peripheral nerve sheath tumors (zMPNST) [58]. Albeit a rare finding in *lrrc50^Hu255h^* zebrafish, these tumors typically do not occur in wild-type zebrafish and might therefore potentially represent an alternative *lrrc50* associated tumor type. Scale bars; 50 µm. (B) We identified a tumor of somatic tissue (undetermined pathology, putative zMPNST) located in the testes of one zebrafish, unlike all other TGCTs described in this manuscript. Scale bars; 50 µm. (C) Heterozygote *lrrc50* females (n = 11) do not show gonadal abnormalities; ovaries were isolated simultaneously with male testes/TGCT at an age of 30 months. Scale bars; 50 µm.
(TIF)

Figure S3 Zebrafish spermatogenesis and *lrrc50^Hu255h^* testicular hyperplasia. (A) The various stages of spermatogenesis in zebrafish can be morphologically distinguished and are indicated in a section from wild type zebrafish. Spermatogonial stem cells (SPG) that commit to spermatogenesis from clusters of paired (SPG_paired) and aligned spermatogonia (SPG_al) by mitotic divisions. All differentiated germ cells remain connected via stabilised intracellular bridges that allow the shared use of cytoplasmic components. This elegant mechanism is essential to synchronise collective mitosis, meiosis, differentiation and apoptosis of these clusters of cells. Next, SPG_al collectively differentiate into primary spermatocytes (PS), reducing cytoplasmic volume and severely modifying nuclear structure. Meiosis is the next step of differentiation, and occurs via the first (MI) and second (MII) meiotic divisions, forming haploid cells, these cells are known as secondary spermatocytes (SS). These cells contain one copy of the genome and need no further divisions; instead, these cells differentiate into spermatids (ST) that eventually form mature sperm. Scale bars; 100 µm. (B) Wild-type and hyperplastic testes of heterozygote *lrrc50^Hu255h^* zebrafish. A total of three have been identified, all showing moderate to extreme increases in testes volume, however upon histology we do not observe the typical increase in early germ cells as observed in tumors. Scale bars; 50 µm.
(TIF)

Figure S4 Zebrafish *lrrc50^Hu255h^* tumor proliferation and genotyping various stages of tumor progression. (A) Quantification of single stem cell proliferation from *phospho*-HistoneH3 staining; *lrrc50*+/− (T; tumors, n = 7), wt (n = 5) and age-matched *vhl*+/− (n = 4). Statistical analysis was performed using a non-parametric Mann-Whitney test at *P*<0.05. (B) Analysis of the tumor progression, defined by SPG content determined by α-Ziwi IHC and morphologically identifiable sperm content, correlates with increased biallelic loss indicated by tumor genotypes. Scale bars; 50 µm.
(TIF)

Figure S5 Detailed characterization of human LRRC50. (A) Adapted image from a report by Muto *et al.* [26] which describes a group of LRR containing proteins that all share localization to the centrosome. Based on the intracellular distribution of LRRC50 described in this manuscript and the presence of six Leucine Rich Repeats as well as a Coiled-Coil domain, we propose LRRC50 to be an additional member of this subgroup. (B) LRRC50 localization resembles that of the group of perichromosomal sheath proteins [27]. A detailed description of the dynamic LRRC50 localization to chromosomes during the cell cycle is provided. Additionally, continuous association of LRRC50 with the centrosomes and localization to the midbody can be appreciated. We used Dapi (blue), α-LRRC50 ab75163 (green) and α-acetylated-α-Tubulin unless otherwise indicated. *Nucleolus*; in interphase cells, LRRC50 localizes to structures in the nucleus. We show these structures to be nucleoli based on co-localization with ectopically expressed mybbp1A-RFP (red), which is an established nucleolar marker [59]. *G2*; cells that are in the G2 phase of the cell cycle show increased expression of LRRC50 in both nucleoli and nucleoplasm. One cell with duplicated centrosomes (using α-γ-Tubulin in red) can be observed, indicating this cell to be in the G2 phase. The neighbouring cell has no duplicated centrosomes and appears to be in interphase. Expression levels are generally lower, supported by the *LRRC50* mRNA expression shown in Figure 5E and Figure S6C, and localization is confined to nucleoli. *Prophase*; upon completion of chromosome condensation at the G2/M transition, LRRC50 has associated with all foci of condensed chromosomes. *Metaphase and Anaphase*; after nuclear envelope breakdown, LRRC50 remains associated with the chromosomes throughout formation of the metaphase plane and the actual chromosome segregation during anaphase. *Telophase and cytokinesis*; during telophase when condensed chromosomes begin to de-condense, LRRC50 relocalizes from the perichromosomal sheath to a more diffuse and less defined chromosomal location. Followed by a temporal redistribution to the midbody.
(TIF)

Figure S6 Human LRRC50 expression. (A) LRRC50 mRNA is analyzed in a cell cycle dependent fashion (Figure 5E). Samples were harvested from T47D cells double blocked in thymidine or released from this block at indicated time points. FACS analysis using propidium iodide and α-phospho-Histone H3 are used to determine the cell cycle stages. Percentages of cells in [SubN2], [G1], [S], [G2] and [M] phases are indicated. (B) RPE-hTERT cells were serum starved for three consecutive days to initiate cilia formation. Expression of *LRRC50* mRNA is increased accordingly. Error bars present standard deviation. Samples are normalized to day 0 expression levels. (C) Graphical overview of *LRRC50* mRNA and protein expression in various cell cycle dependent stages. *LRRC50* is low during interphase prior to a dramatic up-regulation during early S-phase. Protein expression lags shortly behind based on staining intensities. In mid-S-phase/early G2, mRNA has been reduced to interphase levels, indicating a stringent regulation. Protein levels remain elevated throughout mitosis, before decreasing in interphase. Cells either proceed into a new round of mitosis or cells exit from the cell cycle into G0. *LRRC50* mRNA and protein levels are increased during ciliogenesis as has been described in B and as previously described literature [6], [7], [10], [20]. Taken together, the expression pattern and localization of LRRC50 are highly suggestive of a dual protein function; one role required for ciliary processes, another for a cell cycle related function.
(TIF)

Figure S7 Summarizing overview of described data. LRRC50 (green) normally exhibits various intracellular localizations. Defects in LRRC50 (red) have been previously shown to cause ciliary defects resulting in PCD. Here we show that, through an unknown mechanism likely to involve biallelic inactivation, mutations of *LRRC50* results in the formation of seminomas in zebrafish and man. The identified mutant alleles are shown in red.
(TIF)

Table S1 *LRRC50 in vivo* complementation analysis. Statistics and sample size of *in vivo* complementation analyses (NS, not significant; MO, morpholino; WT, wild-type).
(DOC)

Table S2 Primer sequences used for zebrafish and human genotyping. Amplicons spanning zebrafish *lrrc50* and human *LRRC50* exons were designed using the Primer3 software (http://fokker.wi.mit.edu/primer3/input.htm) described by Rozen S and Skaletsky HJ,. (2000). Bioinformatics Methods and Protocols: *Methods Mol Biol. 132*, 365–386.
(DOC)

## References

[1] Oosterhuis JW, Looijenga LHJ (2005) Testicular germ-cell tumours in a broader perspective. Nat Rev Cancer 5: 210–222 doi:10.1038/nrc1568. 15738984 10.1038/nrc1568

[2] Looijenga LH (2009) Human testicular (non)seminomatous germ cell tumours: the clinical implications of recent pathobiological insights. J Pathol 218: 146–162 doi:10.1002/path.2522. 19253916 10.1002/path.2522

[3] Liu S, Leach SD (2011) Zebrafish Models for Cancer. Annu Rev Pathol Mech Dis 6: 71–93 doi:10.1146/annurev-pathol-011110–130330. 10.1146/annurev-pathol-011110-130330 21261518

[4] Raz E (2003) Primordial germ-cell development: the zebrafish perspective. Nat Rev Genet 4: 690–700 doi:10.1038/nrg1154. 12951570 10.1038/nrg1154

[5] Neumann JC, Dovey JS, Chandler GL, Carbajal L, Amatruda JF (2009) Identification of a Heritable Model of Testicular Germ Cell Tumor in the Zebrafish. Zebrafish 6: 319–327 doi:10.1089/zeb.2009.0613. 20047465 10.1089/zeb.2009.0613 PMC2811880

[6] van Rooijen E, Giles RH, Voest EE, van Rooijen C, Schulte-Merker S, et al (2008) LRRC50, a conserved ciliary protein implicated in polycystic kidney disease. J Am Soc Nephrol 19: 1128–1138 doi:10.1681/ASN.2007080917. 18385425 10.1681/ASN.2007080917 PMC2396934

[7] Freshour J, Yokoyama R, Mitchell DR (2007) Chlamydomonas flagellar outer row dynein assembly protein ODA7 interacts with both outer row and I1 inner row dyneins. Journal of Biological Chemistry 282: 5404–5412 doi:10.1074/jbc.M607509200. 17194703 10.1074/jbc.M607509200 PMC3321484

[8] Loges NT, Olbrich H, Becker-Heck A, HAffner K, Heer A, et al (2009) Deletions and Point Mutations of LRRC50 Cause Primary Ciliary Dyskinesia Due to Dynein Arm Defects. The American Journal of Human Genetics 1–7 doi:10.1016/j.ajhg.2009.10.018. 10.1016/j.ajhg.2009.10.018 PMC2795801 19944400

[9] Duquesnoy P, Escudier E, Vincensini L, Freshour J, Bridoux A-M, et al (2009) Loss-of-Function Mutations in the Human Ortholog of Chlamydomonas reinhardtii ODA7 Disrupt Dynein Arm Assembly and Cause Primary Ciliary Dyskinesia. The American Journal of Human Genetics 1–7 doi:10.1016/j.ajhg.2009.11.008. 10.1016/j.ajhg.2009.11.008 PMC2790569 19944405

[10] Sullivan-Brown J, Schottenfeld J, Okabe N, Hostetter CL, Serluca FC, et al (2008) Zebrafish mutations affecting cilia motility share similar cystic phenotypes and suggest a mechanism of cyst formation that differs from pkd2 morphants. Developmental Biology 314: 261–275 doi:10.1016/j.ydbio.2007.11.025. 18178183 10.1016/j.ydbio.2007.11.025 PMC2453220

[11] Leal MC, Cardoso ER, Nobrega RH, Batlouni SR, Bogerd J, et al (2009) Histological and Stereological Evaluation of Zebrafish (Danio rerio) Spermatogenesis with an Emphasis on Spermatogonial Generations. Biology of Reproduction 81: 177–187 doi:10.1095/biolreprod.109.076299. 19339708 10.1095/biolreprod.109.076299

[12] Moore JL, Rush LM, Breneman C, Mohideen MAPK, Cheng KC (2006) Zebrafish Genomic Instability Mutants and Cancer Susceptibility. Genetics 174: 585–600 doi:10.1534/genetics.106.059386. 16888336 10.1534/genetics.106.059386 PMC1602069

[13] Greenbaum M, Ma L, Matzuk M (2007) Conversion of midbodies into germ cell intercellular bridges. Developmental Biology 305: 389–396 doi:10.1016/j.ydbio.2007.02.025. 17383626 10.1016/j.ydbio.2007.02.025 PMC2717030

[14] Qiao DD, Zeeman A-MA, Deng WW, Looijenga LHJL, Lin HH (2002) Molecular characterization of hiwi, a human member of the piwi gene family whose overexpression is correlated to seminomas. Oncogene 21: 3988–3999 doi:10.1038/sj.onc.1205505. 12037681 10.1038/sj.onc.1205505

[15] Houwing S, Kamminga LM, Berezikov E, Cronembold D, Girard A, et al (2007) A role for Piwi and piRNAs in germ cell maintenance and transposon silencing in Zebrafish. Cell 129: 69–82 doi:10.1016/j.cell.2007.03.026. 17418787 10.1016/j.cell.2007.03.026

[16] Neumann JC, Lillard K, Damoulis V, Amatruda JF (2011) Zebrafish Models of Germ Cell Tumor. Third Edition. Elsevier Inc. 24 pp. doi:10.1016/B978-0-12-381320-6.00001-1. 10.1016/B978-0-12-381320-6.00001-1 PMC3932324 21951524

[17] Greene MH, Kratz CP, Mai PL, Mueller C, Peters JA, et al (2010) Familial testicular germ cell tumors in adults: 2010 summary of genetic risk factors and clinical phenotype. Endocrine Related Cancer 17: R109–R121 doi:10.1677/ERC-09-0254. 20228134 10.1677/ERC-09-0254 PMC3101798

[18] Adzhubei IA, Schmidt S, Peshkin L, Ramensky VE, Gerasimova A, et al (2010) A method and server for predicting damaging missense mutations. Nat Methods 7: 248–249 doi:10.1038/nmeth0410-248. 20354512 10.1038/nmeth0410-248 PMC2855889

[19] O'Toole JF, Liu Y, Davis EE, Westlake CJ, Attanasio M, et al (2010) Individuals with mutations in XPNPEP3, which encodes a mitochondrial protein, develop a nephronophthisis-like nephropathy. J Clin Invest 120: 791–802 doi:10.1172/JCI40076DS1. 20179356 10.1172/JCI40076 PMC2827951

[20] Fowkes ME, Mitchell DR (1998) The role of preassembled cytoplasmic complexes in assembly of flagellar dynein subunits. Molecular Biology of the Cell 9: 2337–2347. 9725897 10.1091/mbc.9.9.2337 PMC25499

[21] Smith JC, Northey JGB, Garg J, Pearlman RE, Siu KWM (2005) Robust method for proteome analysis by MS/MS using an entire translated genome: demonstration on the ciliome of Tetrahymena thermophila. J Proteome Res 4: 909–919 doi:10.1021/pr050013h. 15952738 10.1021/pr050013h

[22] Gromley A, Yeaman C, Rosa J, Redick S, Chen C-T, et al (2005) Centriolin Anchoring of Exocyst and SNARE Complexes at the Midbody Is Required for Secretory-Vesicle-Mediated Abscission. Cell 123: 75–87 doi:10.1016/j.cell.2005.07.027. 16213214 10.1016/j.cell.2005.07.027

[23] Tsvetkov L, Xu X, Li J, Stern DF (2003) Polo-like kinase 1 and Chk2 interact and co-localize to centrosomes and the midbody. J Biol Chem 278: 8468–8475 doi:10.1074/jbc.M211202200. 12493754 10.1074/jbc.M211202200

[24] Kim JC, Ou YY, Badano JL, Esmail MA, Leitch CC, et al (2005) MKKS/BBS6, a divergent chaperonin-like protein linked to the obesity disorder Bardet-Biedl syndrome, is a novel centrosomal component required for cytokinesis. Journal of Cell Science 118: 1007–1020 doi:10.1242/jcs.01676. 15731008 10.1242/jcs.01676

[25] Smith KR, Kieserman EK, Wang PI, Basten SG, Giles RH, et al (2011) A role for central spindle proteins in cilia structure and function. Cytoskeleton 68: 112–124 Available: http://eutils.ncbi.nlm.nih.gov/entrez/eutils/elink.fcgi?dbfrom=pubmed&id=21246755&retmode=ref&cmd=prlinks. 21246755 10.1002/cm.20498 PMC4089984

[26] Muto Y, Okano Y (2009) CLERC and centrosomal leucine-rich repeat proteins. centeurjbiol 5: 1–10 doi:10.2478/s11535-009-0061-x.

[27] Hooser AA, Yuh P, Heald R (2005) The perichromosomal layer. Chromosoma 114: 377–388 doi:10.1007/s00412-005-0021-9. 16136320 10.1007/s00412-005-0021-9

[28] Plotnikova OV, Golemis EA, Pugacheva EN (2008) Cell Cycle-Dependent Ciliogenesis and Cancer. Cancer Research 68: 2058–2061 doi:10.1158/0008-5472.CAN-07-5838. 18381407 10.1158/0008-5472.CAN-07-5838 PMC2546565

[29] Smolowitz R, Hanley J, Richmond H (2002) A three-year retrospective study of abdominal tumors in zebrafish maintained in an aquatic laboratory animal facility. Biol Bull 203: 265–266. 12414614 10.2307/1543433

[30] Kent ML, Spitsbergen JM, Matthews JM, Fournie JW, Murray KN, et al. (2012). Diseases of Zebrafish in Research Facilities, Zebrafish International Resource Center. Available: http://zebrafish.org/zirc/health/diseaseManual.php. Accessed 28 December 2012.

[31] Amsterdam A, Lai K, Komisarczuk AZ, Becker TS, Bronson RT, et al (2009) Zebrafish Hagoromo mutants up-regulate fgf8 postembryonically and develop neuroblastoma. Molecular Cancer Research 7: 841–850 doi:10.1158/1541-7786.MCR-08-0555. 19531571 10.1158/1541-7786.MCR-08-0555 PMC2744123

[32] Feitsma H, Cuppen E (2008) Zebrafish as a Cancer Model. Molecular Cancer Research 6: 685–694 doi:10.1158/1541-7786.MCR-07-2167. 18505914 10.1158/1541-7786.MCR-07-2167

[33] Amatruda JF, Patton EE (2008) Genetic models of cancer in zebrafish. Int Rev Cell Mol Biol 271: 1–34 doi:10.1016/S1937-6448(08)01201-X. 19081540 10.1016/S1937-6448(08)01201-X

[34] Neumann JC, Chandler GL, Damoulis VA, Fustino NJ, Lillard K, et al (2011) Mutation in the type IB bone morphogenetic protein receptor Alk6b impairs germ-cell differentiation and causes germ-cell tumors in zebrafish. Proceedings of the National Academy of Sciences 108: 13153–13158 doi:10.1073/pnas.1102311108. 10.1073/pnas.1102311108 PMC3156187 21775673

[35] Horwich A, Shipley J, Huddart R (2006) Testicular germ-cell cancer. Lancet 367: 754–765 doi:10.1016/S0140-6736(06)68305-0. 16517276 10.1016/S0140-6736(06)68305-0

[36] Leigh MW, Pittman JE, Carson JL, Ferkol TW, Dell SD, et al (2009) Clinical and genetic aspects of primary ciliary dyskinesia/Kartagener syndrome. Genetics in Medicine 11: 473–487 doi:10.1097/GIM.0b013e3181a53562. 19606528 10.1097/GIM.0b013e3181a53562 PMC3739704

[37] Fliegauf M, Olbrich H, Horvath J, Wildhaber JH, Zariwala MA, et al (2005) Mislocalization of DNAH5 and DNAH9 in respiratory cells from patients with primary ciliary dyskinesia. Am J Respir Crit Care Med 171: 1343–1349 doi:10.1164/rccm.200411-1583OC. 15750039 10.1164/rccm.200411-1583OC PMC2718478

[38] Wood LD, Parsons DW, Jones S, Lin J, Sjöblom T, et al (2007) The genomic landscapes of human breast and colorectal cancers. Science 318: 1108–1113 doi:10.1126/science.1145720. 17932254 10.1126/science.1145720

[39] Yuan K, Frolova N, Xie Y, Wang D, Cook L, et al (2010) Primary cilia are decreased in breast cancer: analysis of a collection of human breast cancer cell lines and tissues. J Histochem Cytochem 58: 857–870 doi:10.1369/jhc.2010.955856. 20530462 10.1369/jhc.2010.955856 PMC2942739

[40] Händel M, Schulz S, Stanarius A, Schreff M, Erdtmann-Vourliotis M, et al (1999) Selective targeting of somatostatin receptor 3 to neuronal cilia. Neuroscience 89: 909–926. 10199624 10.1016/s0306-4522(98)00354-6

[41] Baou N, Bouras M, Droz JP, Benahmed M, Krantic S (2000) Evidence for a selective loss of somatostatin receptor subtype expression in male germ cell tumors of seminoma type. Carcinogenesis 21: 805–810. 10753219 10.1093/carcin/21.4.805

[42] Neugebauer JM, Amack JD, Peterson AG, Bisgrove BW, Yost HJ (2009) FGF signalling during embryo development regulates cilia length in diverse epithelia. Nature 458: 651–654 doi:10.1038/nature07753. 19242413 10.1038/nature07753 PMC2688717

[43] Suzuki K, Tokue A, Kamiakito T, Kuriki K, SAITO K, et al (2001) Predominant expression of fibroblast growth factor (FGF) 8, FGF4, and FGF receptor 1 in nonseminomatous and highly proliferative components of testicular germ cell tumors. Virchows Arch 439: 616–621 doi:10.1007/s004280100437. 11764380 10.1007/s004280100437

[44] Palumbo C, van Roozendaal K, Gillis AJ, van Gurp RH, de Munnik H, et al (2002) Expression of the PDGF alpha-receptor 1.5 kb transcript, OCT-4, and c-KIT in human normal and malignant tissues. Implications for the early diagnosis of testicular germ cell tumours and for our understanding of regulatory mechanisms. J Pathol 196: 467–477 doi:10.1002/path.1064. 11920744 10.1002/path.1064

[45] Schneider L, Clement CA, Teilmann SC, Pazour GJ, Hoffmann EK, et al (2005) PDGFRαα Signaling Is Regulated through the Primary Cilium in Fibroblasts. Current Biology 15: 1861–1866 doi:10.1016/j.cub.2005.09.012. 16243034 10.1016/j.cub.2005.09.012

[46] Mans DA, Voest EE, Giles RH (2008) All along the watchtower: Is the cilium a tumor suppressor organelle? BBA - Reviews on Cancer 1786: 114–125 doi:10.1016/j.bbcan.2008.02.002. 18343234 10.1016/j.bbcan.2008.02.002

[47] Gómez García EB, Knoers NVAM (2009) Gardner's syndrome (familial adenomatous polyposis): a cilia-related disorder. Lancet Oncol 10: 727–735 doi:10.1016/S1470-2045(09)70167-6. 19573802 10.1016/S1470-2045(09)70167-6

[48] Oosterhuis JW, Looijenga LHJ (2012) Current views on the pathogenesis of testicular germcell tumours and perspectives for future research:Highlights of the 5th Copenhagen Workshop on Carcinomain situ and Cancer of the Testis. 1–10. 10.1034/j.1600-0463.2003.1110131.x 12752274

[49] Rapley EA, Turnbull C, Olama Al AA, Dermitzakis ET, Linger R, et al (2009) A genome-wide association study of testicular germ cell tumor. Nature Publishing Group 41: 807–810 doi:10.1038/ng.394. 10.1038/ng.394 PMC2871592 19483681

[50] Kanetsky PA, Mitra N, Vardhanabhuti S, Li M, Vaughn DJ, et al (2009) Common variation in KITLG and at 5q31.3 predisposes to testicular germ cell cancer. Nature Publishing Group 41: 811–815 doi:10.1038/ng.393. 10.1038/ng.393 PMC2865677 19483682

[51] Rapley EA, Nathanson KL (2010) Predisposition alleles for testicular germ cell tumour. Current Opinion in Genetics & Development 1–6 doi:10.1016/j.gde.2010.02.006. 20303738 10.1016/j.gde.2010.02.006

[52] Olie RA, Looijenga LH, Boerrigter L, Top B, Rodenhuis S, et al (1995) N- and KRAS mutations in primary testicular germ cell tumors: incidence and possible biological implications. Genes Chromosomes Cancer 12: 110–116. 7535083 10.1002/gcc.2870120205

[53] Vladušić T, Hrašćan R, Vrhovac I, Krušlin B, Gamulin M, et al (2010) Loss of heterozygosity of selected tumor suppressor genes in human testicular germ cell tumors. Pathology Research and Practice 206: 163–167 Available: http://linkinghub.elsevier.com/retrieve/pii/S0344033809002684. 10.1016/j.prp.2009.10.007 20092957

[54] Looijenga LHJ (2006) Genomic and Expression Profiling of Human Spermatocytic Seminomas: Primary Spermatocyte as Tumorigenic Precursor and DMRT1 as Candidate Chromosome 9 Gene. Cancer Research 66: 290–302 doi:10.1158/0008-5472.CAN-05-2936. 16397242 10.1158/0008-5472.CAN-05-2936

[55] van Rooijen E, Voest EE, Logister I, Korving J, Schwerte T, et al (2009) Zebrafish mutants in the von Hippel-Lindau tumor suppressor display a hypoxic response and recapitulate key aspects of Chuvash polycythemia. Blood 113: 6449–6460 doi:10.1182/blood-2008-07-167890. 19304954 10.1182/blood-2008-07-167890

[56] van der Ven K, Nguyen TQ, Goldschmeding R (2007) Immunofluorescence on proteinase XXIV-digested paraffin sections. Kidney Int 72: 896 doi:10.1038/sj.ki.5002495. 17882246 10.1038/sj.ki.5002495

[57] Davis EE, Zhang Q, Liu Q, Diplas BH, Davey LM, et al (2011) TTC21B contributes both causal and modifying alleles across the ciliopathy spectrum. Nat Genet 43: 189–196 doi:10.1038/ng.756. 21258341 10.1038/ng.756 PMC3071301

[58] Berghmans S, Murphey RD, Wienholds E, Neuberg D, Kutok JL, et al (2005) tp53 mutant zebrafish develop malignant peripheral nerve sheath tumors. Proc Natl Acad Sci U S A 102: 407–412 doi:10.1073/pnas.0406252102. 15630097 10.1073/pnas.0406252102 PMC544293

[59] Keough RA, Macmillan EM, Lutwyche JK, Gardner JM, Tavner FJ, et al (2003) Myb-binding protein 1a is a nucleocytoplasmic shuttling protein that utilizes CRM1-dependent and independent nuclear export pathways. Experimental Cell Research 289: 108–123 doi:10.1016/S0014-4827(03)00262-3. 12941609 10.1016/s0014-4827(03)00262-3

