# Supplementary figures and images for: Mutations in LRRC50 Predispose Zebrafish and Humans to Seminomas
Source: PLoS Genet. 2013 Apr 11;9(4):e1003384. doi: 10.1371/journal.pgen.1003384 (PMC3627517; doi:10.1371/journal.pgen.1003384)

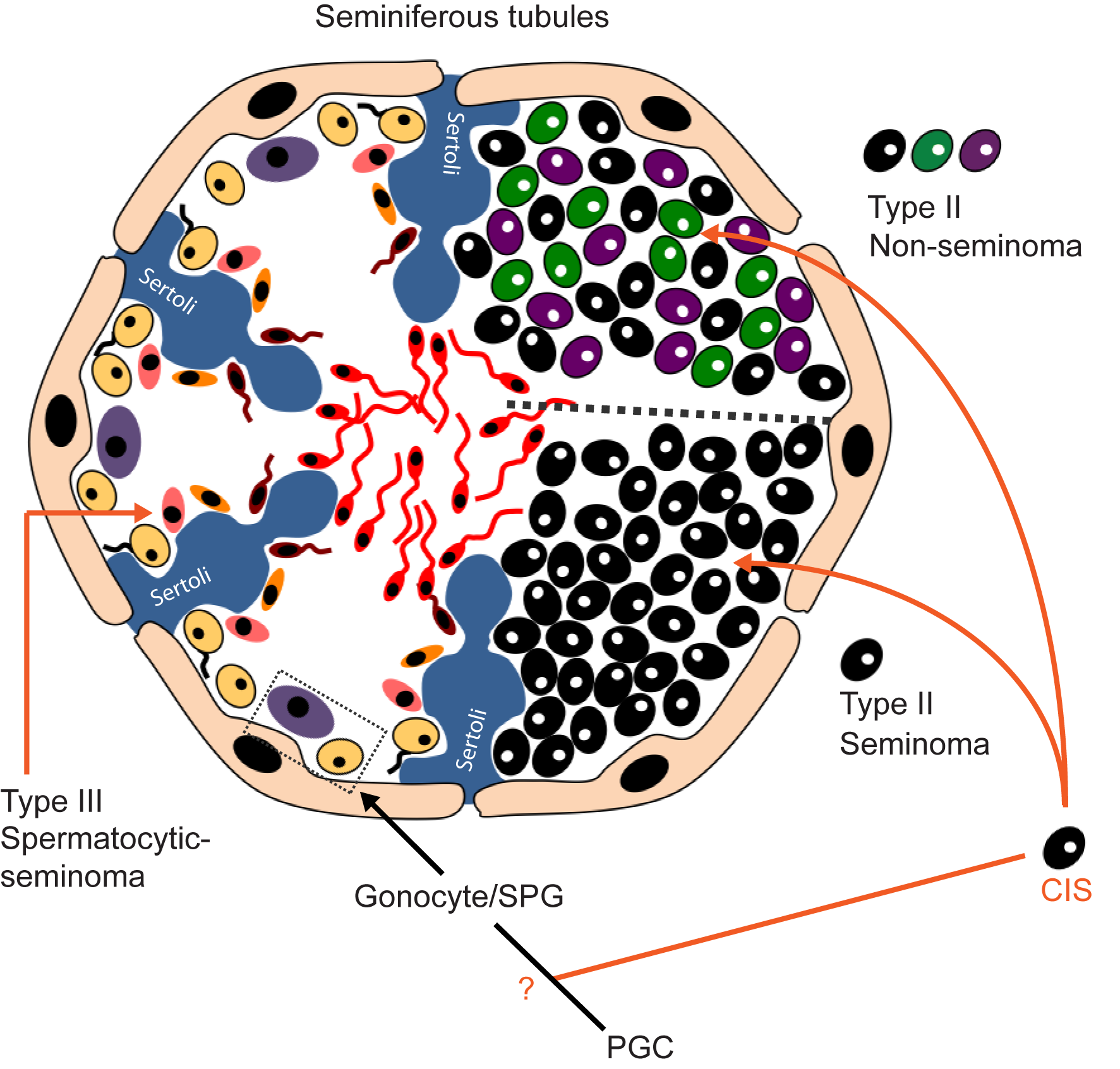

Supplement: Figure S1 — Schematic overview of human TGCT development. Schematic representation of human testicular germ cell development (black lines) giving rise to the known tumor (orange lines) pathologies [1]. Type I GCT are not indicated, but are derived from early stages of embryonic stem cells with different characteristics. Type II GCT commonly arise from transformed, intrinsically pluripotent PGC/gonocytes forming an oncogenic counterpart known as a carcinoma in situ (CIS) cell. The Type II tumors can be seminomas or non-seminomas, the latter forming a more complex pathology consisting of multiple tumorigenic cell types that display characteristics of undifferentiated stem cells, as well as differentiated derivatives (the latter similarly observed in type I GCTs). In normal development, gonocytes establish the germ cell lineage in the seminiferous tubules and differentiate to spermatogonial stem cells that at some point become committed to spermatogenesis. In the seminiferous tubules, the Sertoli cell supports and co-regulates germ cell differentiation and development. Type III GCT are usually recovered from old-aged men and are thought to arise from spermatogonia, i.e., germ cell that lost their embryonic characteristics, committed to spermatogenesis, which is a more differentiated stage of spermatogenesis. (TIF) [file pgen.1003384.s001.tif]

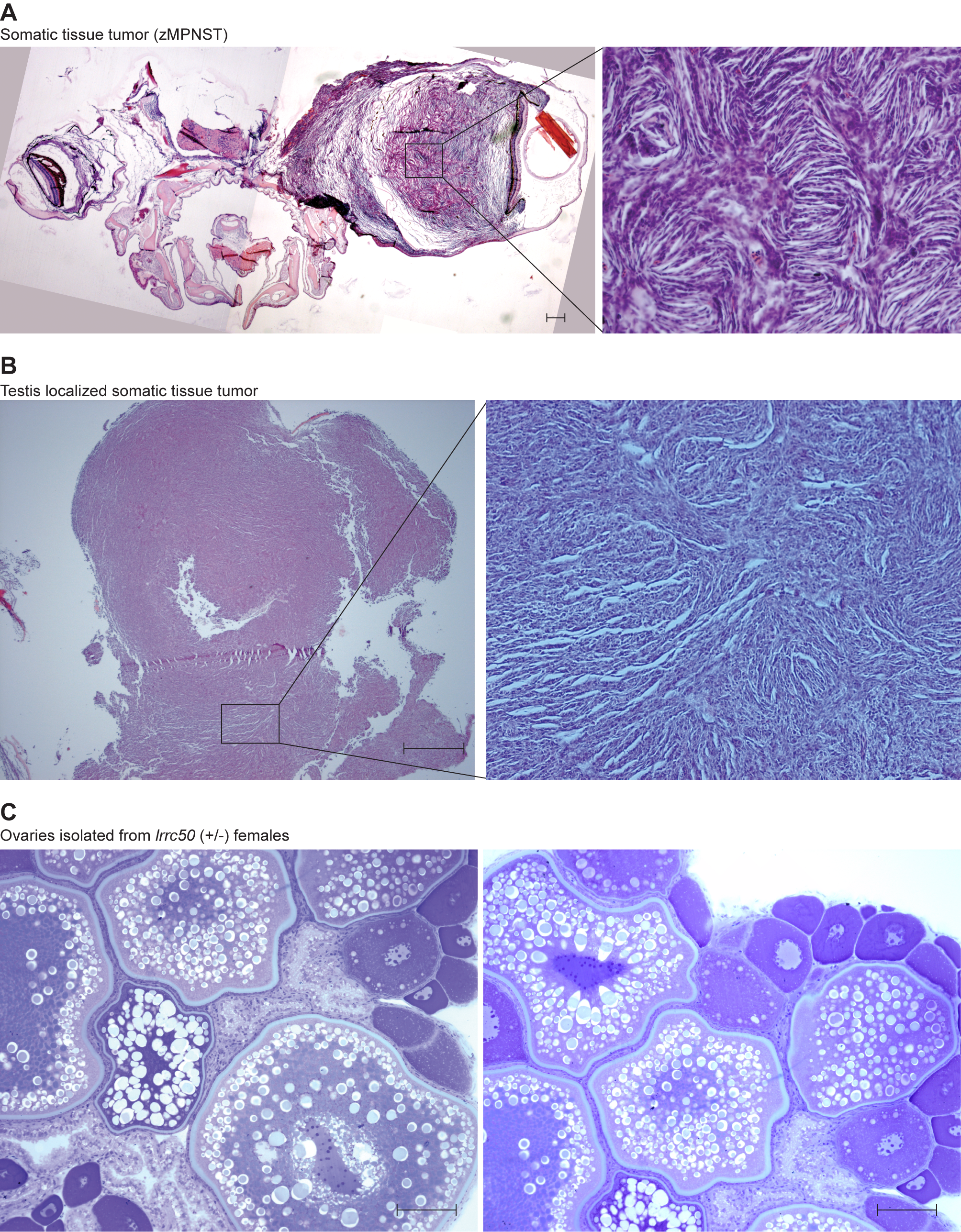

Supplement: Figure S2 — Non-TGCT zebrafish lrrc50Hu255h tumors and female gonad. (A) One fish was identified bearing a large tumor (merge of two images) located proximal to the brain, histologically resembling zebrafish malignant peripheral nerve sheath tumors (zMPNST) [58]. Albeit a rare finding in lrrc50Hu255h zebrafish, these tumors typically do not occur in wild-type zebrafish and might therefore potentially represent an alternative lrrc50 associated tumor type. Scale bars; 50 µm. (B) We identified a tumor of somatic tissue (undetermined pathology, putative zMPNST) located in the testes of one zebrafish, unlike all other TGCTs described in this manuscript. Scale bars; 50 µm. (C) Heterozygote lrrc50 females (n = 11) do not show gonadal abnormalities; ovaries were isolated simultaneously with male testes/TGCT at an age of 30 months. Scale bars; 50 µm. (TIF) [file pgen.1003384.s002.tif]

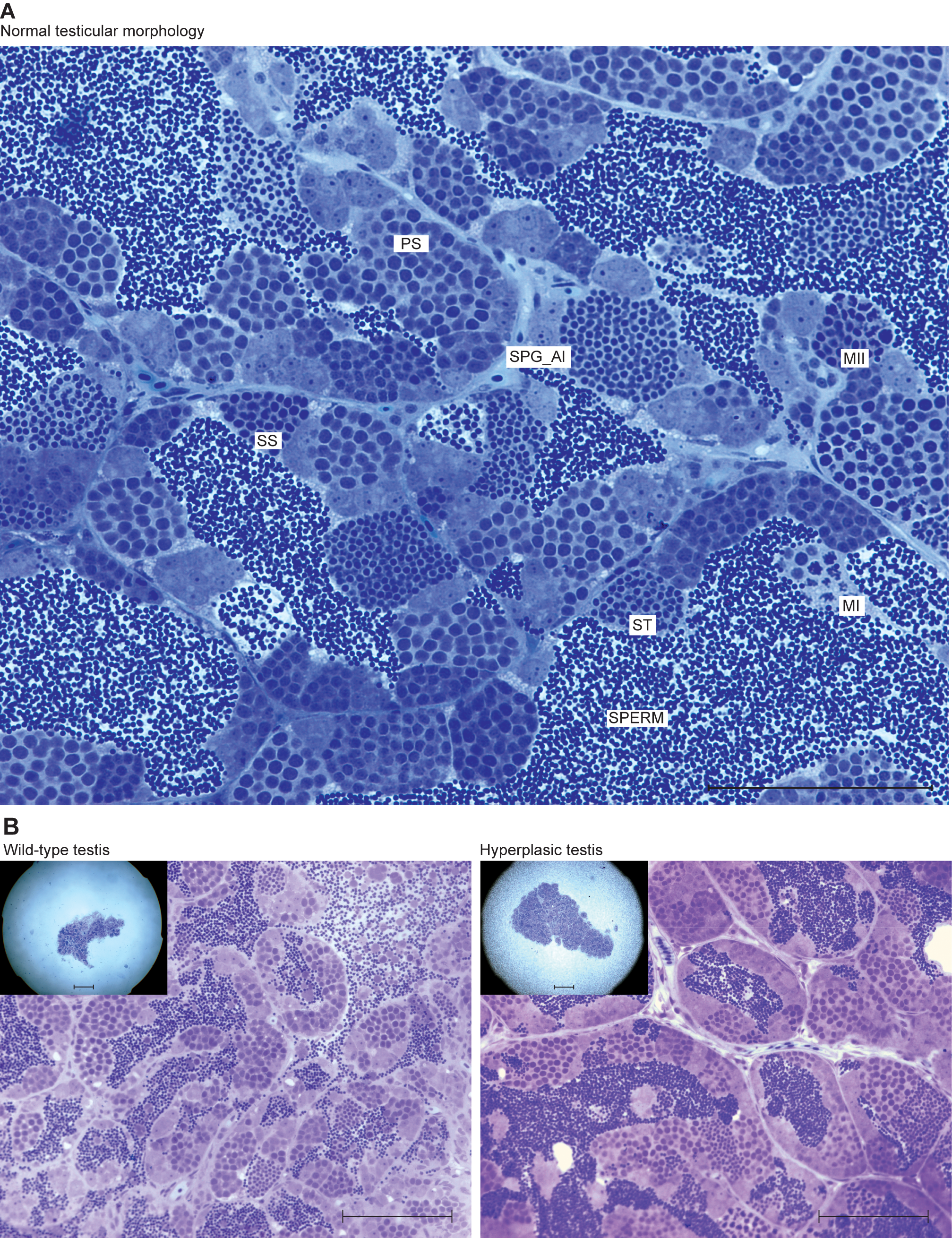

Supplement: Figure S3 — Zebrafish spermatogenesis and lrrc50Hu255h testicular hyperplasia. (A) The various stages of spermatogenesis in zebrafish can be morphologically distinguished and are indicated in a section from wild type zebrafish. Spermatogonial stem cells (SPG) that commit to spermatogenesis from clusters of paired (SPG_paired) and aligned spermatogonia (SPG_al) by mitotic divisions. All differentiated germ cells remain connected via stabilised intracellular bridges that allow the shared use of cytoplasmic components. This elegant mechanism is essential to synchronise collective mitosis, meiosis, differentiation and apoptosis of these clusters of cells. Next, SPG_al collectively differentiate into primary spermatocytes (PS), reducing cytoplasmic volume and severely modifying nuclear structure. Meiosis is the next step of differentiation, and occurs via the first (MI) and second (MII) meiotic divisions, forming haploid cells, these cells are known as secondary spermatocytes (SS). These cells contain one copy of the genome and need no further divisions; instead, these cells differentiate into spermatids (ST) that eventually form mature sperm. Scale bars; 100 µm. (B) Wild-type and hyperplastic testes of heterozygote lrrc50Hu255h zebrafish. A total of three have been identified, all showing moderate to extreme increases in testes volume, however upon histology we do not observe the typical increase in early germ cells as observed in tumors. Scale bars; 50 µm. (TIF) [file pgen.1003384.s003.tif]

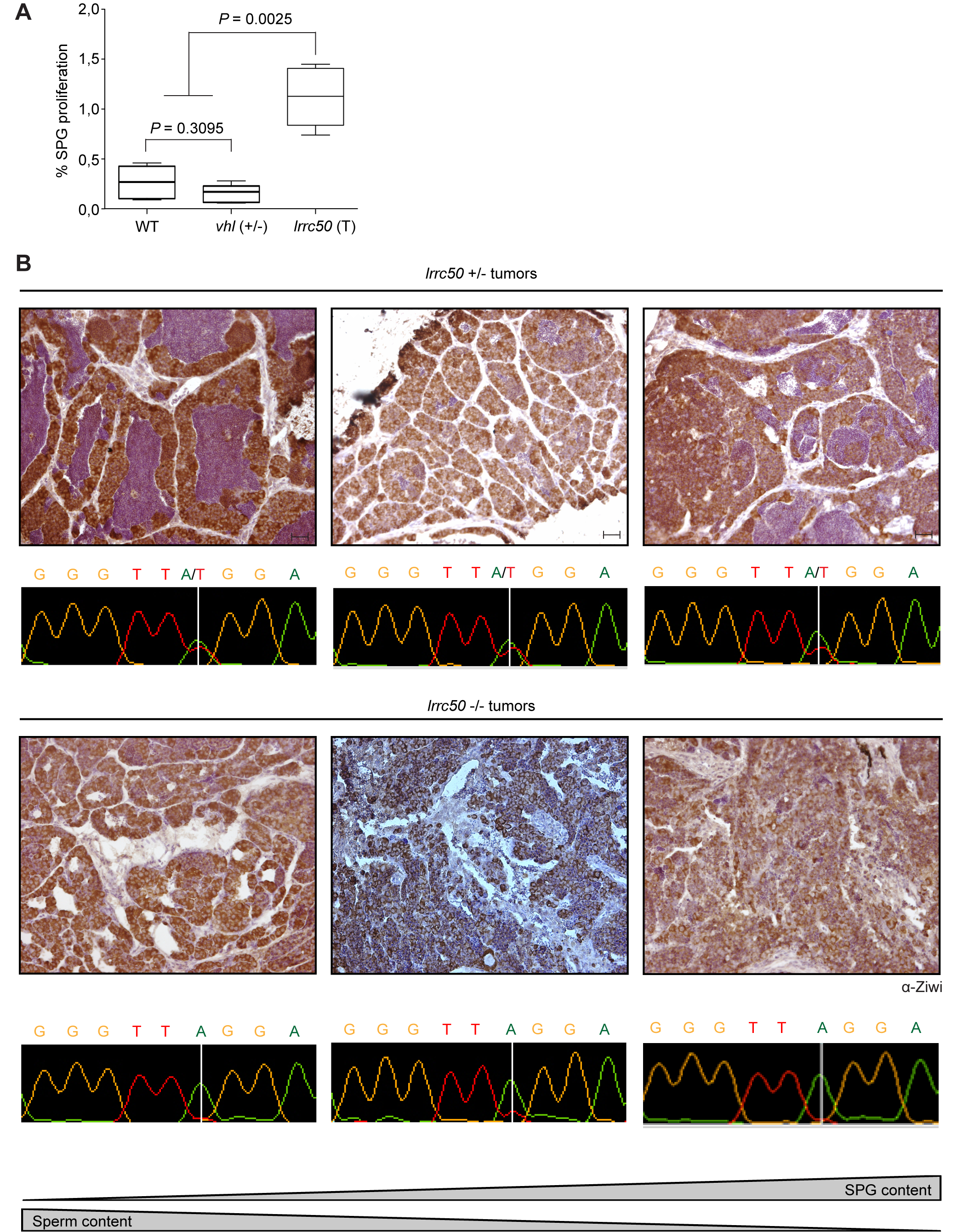

Supplement: Figure S4 — Zebrafish lrrc50Hu255h tumor proliferation and genotyping various stages of tumor progression. (A) Quantification of single stem cell proliferation from phospho-HistoneH3 staining; lrrc50+/− (T; tumors, n = 7), wt (n = 5) and age-matched vhl+/− (n = 4). Statistical analysis was performed using a non-parametric Mann-Whitney test at P<0.05. (B) Analysis of the tumor progression, defined by SPG content determined by α-Ziwi IHC and morphologically identifiable sperm content, correlates with increased biallelic loss indicated by tumor genotypes. Scale bars; 50 µm. (TIF) [file pgen.1003384.s004.tif]

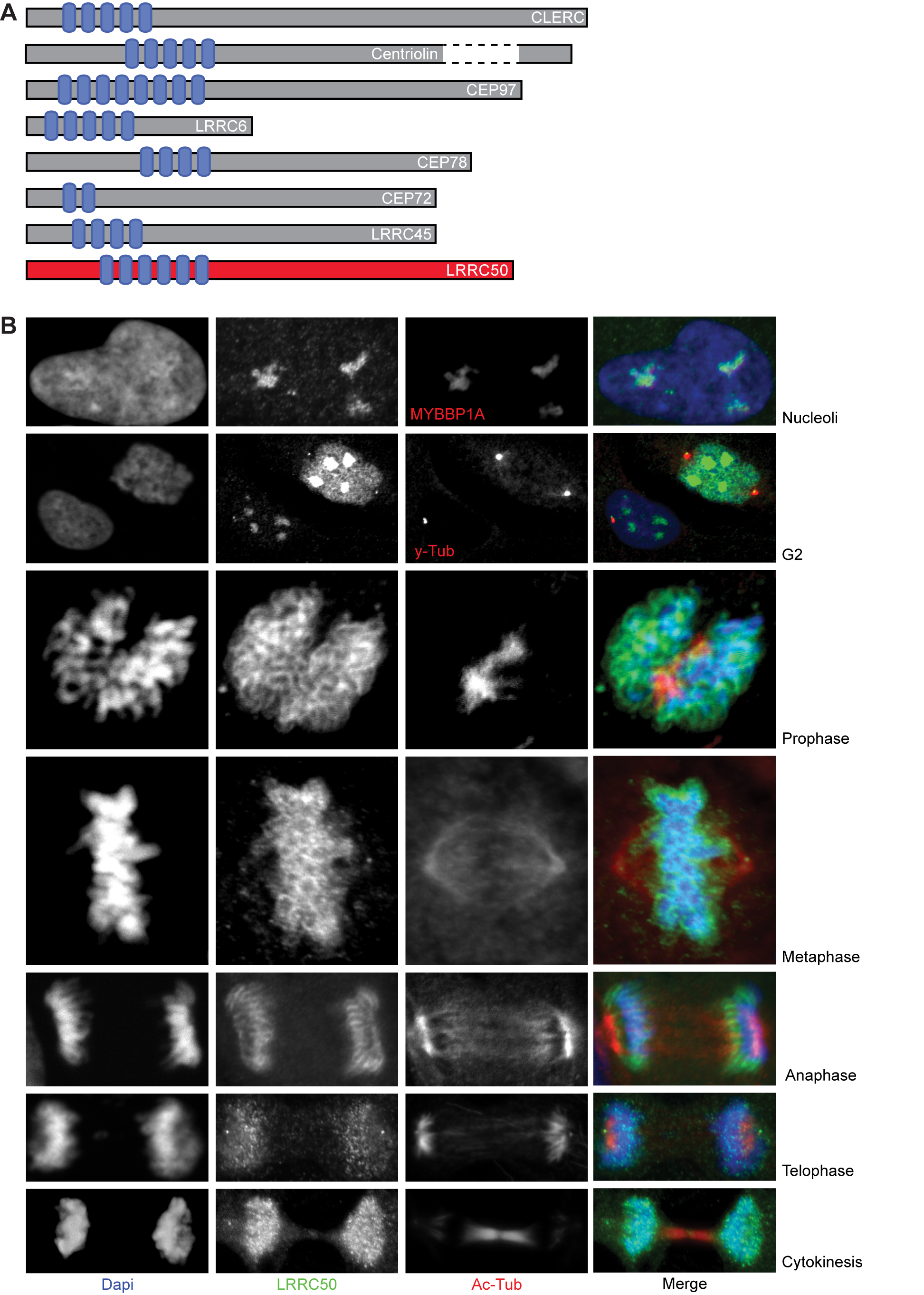

Supplement: Figure S5 — Detailed characterization of human LRRC50. (A) Adapted image from a report by Muto et al. [26] which describes a group of LRR containing proteins that all share localization to the centrosome. Based on the intracellular distribution of LRRC50 described in this manuscript and the presence of six Leucine Rich Repeats as well as a Coiled-Coil domain, we propose LRRC50 to be an additional member of this subgroup. (B) LRRC50 localization resembles that of the group of perichromosomal sheath proteins [27]. A detailed description of the dynamic LRRC50 localization to chromosomes during the cell cycle is provided. Additionally, continuous association of LRRC50 with the centrosomes and localization to the midbody can be appreciated. We used Dapi (blue), α-LRRC50 ab75163 (green) and α-acetylated-α-Tubulin unless otherwise indicated. Nucleolus; in interphase cells, LRRC50 localizes to structures in the nucleus. We show these structures to be nucleoli based on co-localization with ectopically expressed mybbp1A-RFP (red), which is an established nucleolar marker [59]. G2; cells that are in the G2 phase of the cell cycle show increased expression of LRRC50 in both nucleoli and nucleoplasm. One cell with duplicated centrosomes (using α-γ-Tubulin in red) can be observed, indicating this cell to be in the G2 phase. The neighbouring cell has no duplicated centrosomes and appears to be in interphase. Expression levels are generally lower, supported by the LRRC50 mRNA expression shown in Figure 5E and Figure S6C, and localization is confined to nucleoli. Prophase; upon completion of chromosome condensation at the G2/M transition, LRRC50 has associated with all foci of condensed chromosomes. Metaphase and Anaphase; after nuclear envelope breakdown, LRRC50 remains associated with the chromosomes throughout formation of the metaphase plane and the actual chromosome segregation during anaphase. Telophase and cytokinesis; during telophase when condensed chromosomes begin to de-condense, LRR [file pgen.1003384.s005.tif]

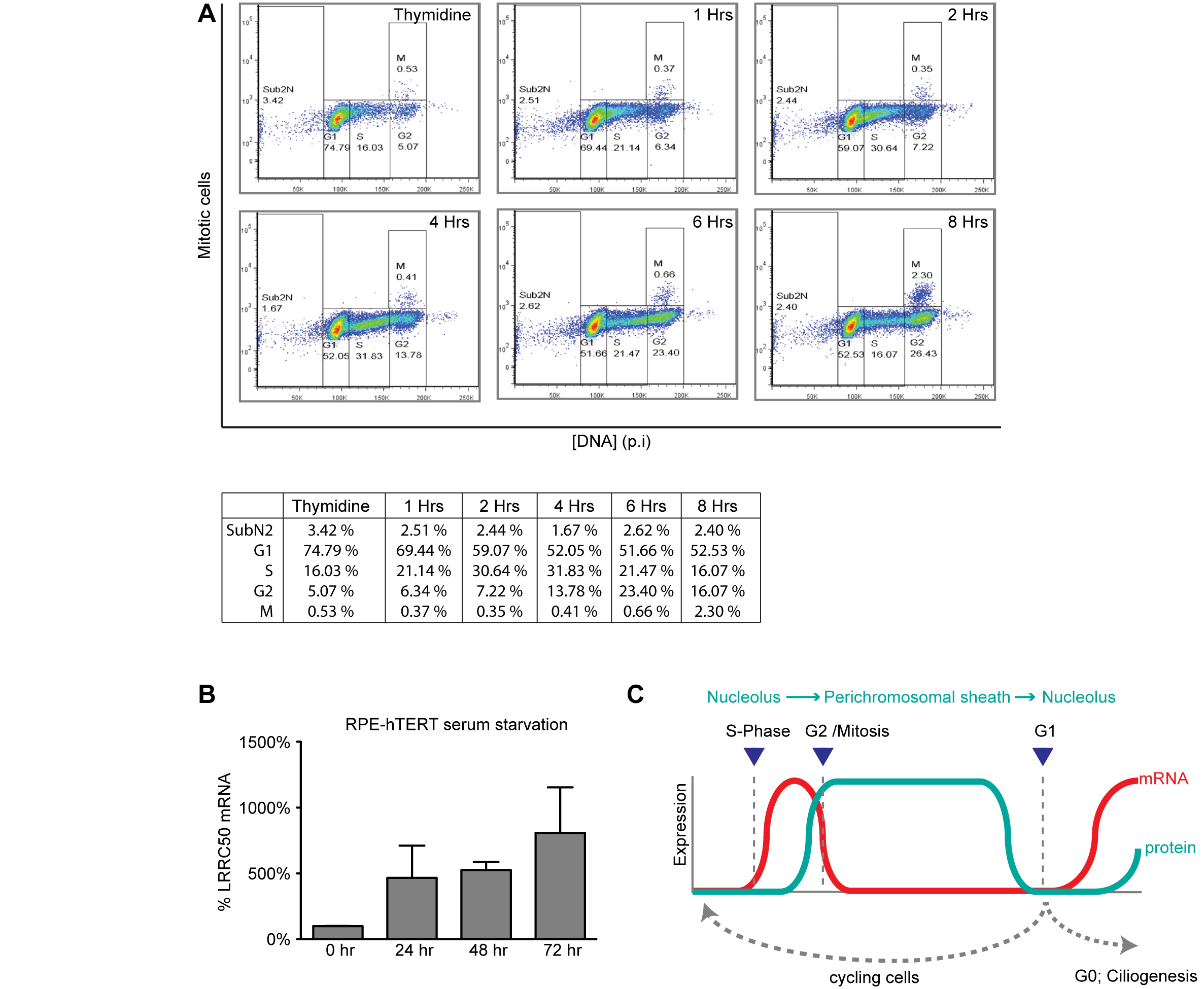

Supplement: Figure S6 — Human LRRC50 expression. (A) LRRC50 mRNA is analyzed in a cell cycle dependent fashion (Figure 5E). Samples were harvested from T47D cells double blocked in thymidine or released from this block at indicated time points. FACS analysis using propidium iodide and α-phospho-Histone H3 are used to determine the cell cycle stages. Percentages of cells in [SubN2], [G1], [S], [G2] and [M] phases are indicated. (B) RPE-hTERT cells were serum starved for three consecutive days to initiate cilia formation. Expression of LRRC50 mRNA is increased accordingly. Error bars present standard deviation. Samples are normalized to day 0 expression levels. (C) Graphical overview of LRRC50 mRNA and protein expression in various cell cycle dependent stages. LRRC50 is low during interphase prior to a dramatic up-regulation during early S-phase. Protein expression lags shortly behind based on staining intensities. In mid-S-phase/early G2, mRNA has been reduced to interphase levels, indicating a stringent regulation. Protein levels remain elevated throughout mitosis, before decreasing in interphase. Cells either proceed into a new round of mitosis or cells exit from the cell cycle into G0. LRRC50 mRNA and protein levels are increased during ciliogenesis as has been described in B and as previously described literature [6], [7], [10], [20]. Taken together, the expression pattern and localization of LRRC50 are highly suggestive of a dual protein function; one role required for ciliary processes, another for a cell cycle related function. (TIF) [file pgen.1003384.s006.tif]

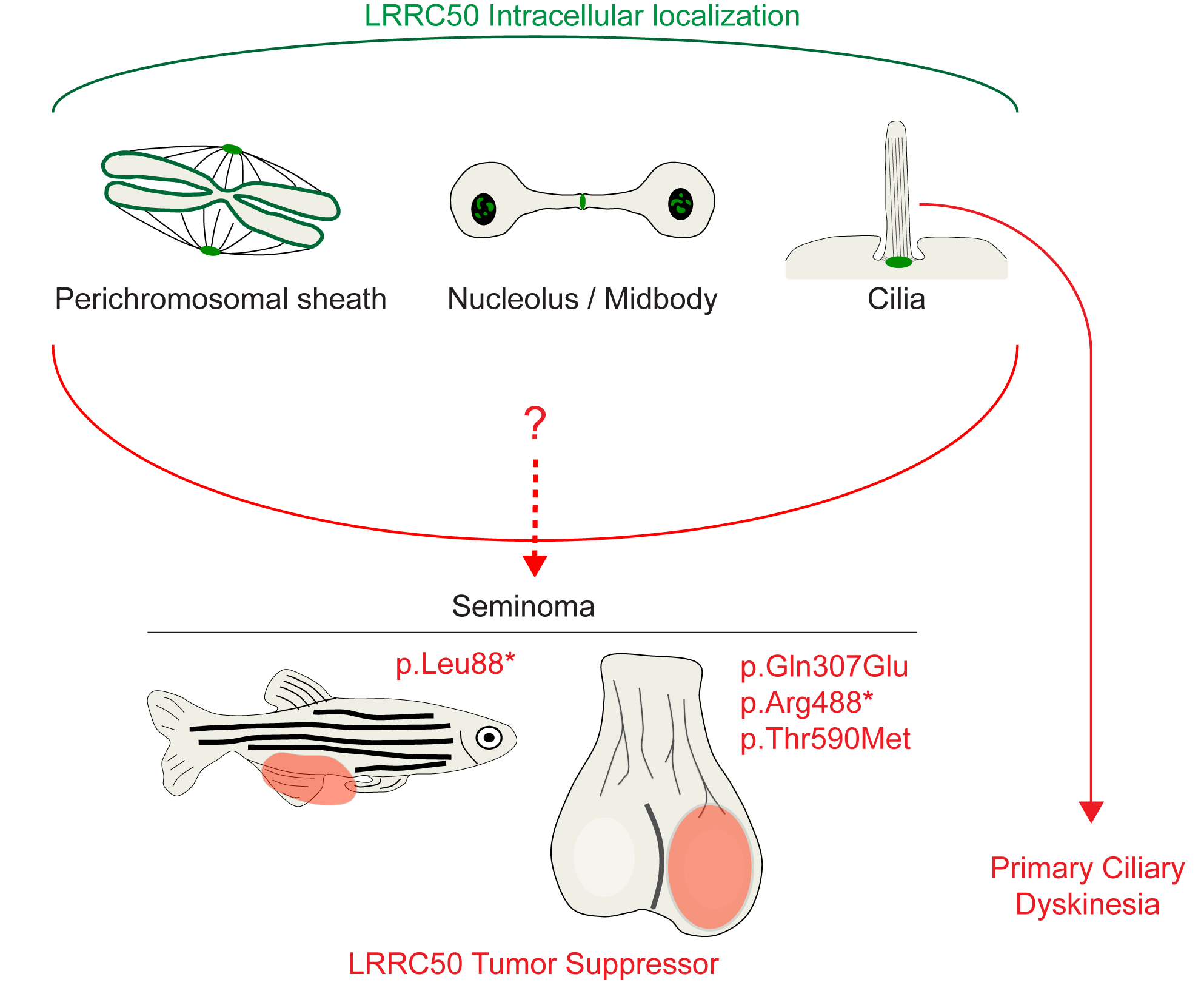

Supplement: Figure S7 — Summarizing overview of described data. LRRC50 (green) normally exhibits various intracellular localizations. Defects in LRRC50 (red) have been previously shown to cause ciliary defects resulting in PCD. Here we show that, through an unknown mechanism likely to involve biallelic inactivation, mutations of LRRC50 results in the formation of seminomas in zebrafish and man. The identified mutant alleles are shown in red. (TIF) [file pgen.1003384.s007.tif]
