# Supplementary material for: Mutations in LRRC50 Predispose Zebrafish and Humans to Seminomas
Source: PLoS Genet. 2013 Apr 11;9(4):e1003384. doi: 10.1371/journal.pgen.1003384 (PMC3627517; doi:10.1371/journal.pgen.1003384)
Supplement: Table S1 — LRRC50 in vivo complementation analysis. Statistics and sample size of in vivo complementation analyses (NS, not significant; MO, morpholino; WT, wild-type). (DOC) [file pgen.1003384.s008.doc]

**Table S1. *LRRC50 in vivo* complementation analysis**

|  |  | **vs. wt rescue** | | | **vs. MO** | | |
| --- | --- | --- | --- | --- | --- | --- | --- |
| Injection | n= | chi-square | P-val | effect | chi-square | P-val | effect |
| Controls | 138 |  |  |  |  |  |  |
| *lrrc50* MO | 88 |  |  |  |  |  |  |
| *lrrc50* MO + LRRC50 WT mRNA | 96 |  |  |  | 58.712 | <0.0001 | + |
| *lrrc50* MO + LRRC50 Q307E mRNA | 158 | 324.011 | <0.0001 | - | 2.863 | 0.239 | NS |
| *lrrc50* MO + LRRC50 T590M mRNA | 122 | 217.1 | <0.0001 | - | 1.164 | 0.5587 | NS |
|  |  | **vs. wt mRNA** | | | **vs. MO** | | |
|  |  | chi-square | P-val | effect | chi-square | P-val | effect |
| *LRRC50* WT mRNA | 110 |  |  |  | 56.715 | <0.0001 | + |
| *LRRC50* Q307E mRNA | 110 | 3.589 | 0.1662 | NS | 105.281 | <0.0001 | + |
| *LRRC50* T590M mRNA | 88 | 0.122 | 0.9407 | NS | 613.822 | <0.0001 | + |

Statistics and sample size of *in vivo* complementation analyses (NS, not significant; MO, morpholino; WT, wild-type).
