# Supplementary material for: Mutations in LRRC50 Predispose Zebrafish and Humans to Seminomas
Source: PLoS Genet. 2013 Apr 11;9(4):e1003384. doi: 10.1371/journal.pgen.1003384 (PMC3627517; doi:10.1371/journal.pgen.1003384)
Supplement: Table S2 — Primer sequences used for zebrafish and human genotyping. Amplicons spanning zebrafish lrrc50 and human LRRC50 exons were designed using the Primer3 software (http://fokker.wi.mit.edu/primer3/input.htm) described by Rozen S and Skaletsky HJ,. (2000). Bioinformatics Methods and Protocols: Methods Mol Biol. 132, 365–386. (DOC) [file pgen.1003384.s009.doc]

**Table S2**: Primer sequences.

| **Primer name** | **Fw (5-3’)** | **Rv (5-3’)** |
| --- | --- | --- |
| Zf_exon_1 | gggACACATTCgTgAgTTTC | ACTgAATAATgCAgCAATgg |
| Zf_exon_2+3 | AgCCAAAgCTgAAggATATg | TACAATggAgAATTAACAgTTTC |
| Zf_exon_4 | TgAATgACACACTTTATCTTCAC | TCTgTAgAgTggACAAgTCg |
| Zf_exon_5+6 | gCACgCTTAATgTCTCAAAC | AgCTTTgTggAgAAAACTgC |
| Zf_exon_7 | AggACACCAgCCCTCTATg | CCAAAgTAAgAgAATgTgAgTgC |
| Zf_exon_8 | CTgTTCATgCCAATTCAAAg | gAAgATCCTAgggAgAATCC |
| Zf_exon_9 | ATCAACCTTTCAgATgAACC | gACACTgCAgTTCACAAAATg |
| Zf_exon_10 | TCACTTggTgAgAAAgCAAg | TATggTAgACgTCCCATTTg |
| Hs_exon_1 | CTggCgAAgAAggAAAgAg | CTgTgAAgATCgggTATgTg |
| Hs_exon_2 | gATggTCATTAACCAAgCTg | CCAgCCTCTATTTCATTATCC |
| Hs_exon_3 | CAggAATggATgTggTAAAg | TTCTgAgAACTAAAggTgATCC |
| Hs_exon_4 | TTGAAGGGACACAGACATTC, | AAAGCTTAGAACATCCCTCAG |
| Hs_exon_5 | ACAggTATgAACCACTgTgC | TGGCATTTCAGATACTCCAG |
| Hs_exon_6 | ggACAggATATTggCACTTC | TAgCCATCAAgCCTATTTCC |
| Hs_exon_7 | CTGATGCTCACTTTGCTTTG | TTAAAGACTGGGTTTGAGAGC |
| Hs_exon_8 | TGTGAGCCCTTGATGTACC | TCCCACAGAGACGTGAGTC |
| Hs_exon_9 | GAGCCCATCTTCACCGTAG | TATCGAGGGTCTCCAAAGTC |
| Hs_exon_10 | AACTAAGGCTGGGTTGACTG | GGAGAGAAACGGTAAACGAC |
| Hs_exon_11 | TGGATGTGGCAACAGAATAC | AGGGTAAGGCTGAGTGACTG |
| Hs_exon_12 | AATTTGGCCTGGACTGAAC | CCCAATCATACACTGACTCG |

Primer sequences used for zebrafish and human genotyping. Amplicons spanning zebrafish *lrrc50* and human *LRRC50* exons were designed using the Primer3 software (http://fokker.wi.mit.edu/primer3/input.htm) [60].
